# Deep learning-based prediction of gene expression from histopathology identifies *NR5A1* as a candidate biomarker and druggable target in high-grade serous ovarian carcinoma

**DOI:** 10.1186/s13048-026-02166-y

**Published:** 2026-06-11

**Authors:** Prakash Lingasamy, Marta Ostrowska-Leśko, Pantelis Tsakalis, Naisarg Patel, Ilias Chamatidis, Sajitha Lulu Sudhakaran, Joanna Kubik, Marcin Bobiński, Nikos D. Lagaros, Andres Salumets, Vijayachitra Modhukur

**Affiliations:** 1 Laboratory of Precision and Nanomedicine, Institute of Biomedicine and Translational Medicine, University of Tartu, Tartu, 50411 Estonia; 2 Celvia CC AS, Tartu, 50411 Estonia; 3 Independent Laboratory of Translational Medicine, Chair of Medical Genetics, Medical University of Lublin, Radziwillowska 11, Lublin, 20-080 Poland; 4 Inferesence(INFS), National Technical University of Athens, Athens, 15780 Greece; 5 Integrative Multiomics Lab, School of Bio Sciences and Technology, Vellore Institute of Technology, Vellore, 632014 Tamil Nadu India; 6 Independent Medical Biology Unit, Medical University of Lublin, Jaczewskiego 8b, Lublin, 20-093 Poland; 7 Division of Obstetrics and Gynaecology, Department of Clinical Science, Intervention and Technology (CLINTEC), Karolinska Institutet, Karolinska University Hospital, Stockholm, 14152 Sweden; 8 Department of Obstetrics and Gynecology, Institute of Clinical Medicine, University of Tartu, Tartu, 50406 Estonia

**Keywords:** High-grade serous ovarian cancer, Computational pathology, Self-supervised learning, Gene expression prediction, Platinum response, Digital pathology, Drug repurposing, Molecular biomarkers, *NR5A1*

## Abstract

**Background:**

High-grade serous ovarian cancer (HGSOC) is the most lethal ovarian cancer subtype, responsible for ~ 70% of ovarian cancer–related deaths and marked by late-stage diagnosis and frequent platinum resistance. Although transcriptomic profiling enables molecular stratification and prediction of therapeutic response; routine clinical use of this approach is limited by cost and logistical constraints. Computational pathology analysis offers a scalable alternative by inferring transcriptional states directly from routine hematoxylin and eosin (H&E) whole-slide images (WSIs).

**Methods:**

Paired H&E WSIs and RNA-sequencing data from the TCGA-OV cohort, including 1,371 diagnostic H&E WSIs retrieved for preprocessing and quality control, were used to develop a self-supervised virtual-transcriptomics framework based on Momentum Contrast v2 (MoCo v2) and multi-output Random Forest regression. Model performance was assessed using patient-level five-fold cross-validation. Candidate genes were evaluated by reverse transcription quantitative polymerase chain reaction (RT-qPCR) in an independent cohort of 10 HGSOC tumors, including 4 platinum responders and 6 non-responders.

**Results:**

The model predicted expression of approximately 6,400 protein-coding genes, achieving a genome-wide mean Pearson correlation of *r* = 0.36, with more than 300 genes showing stronger image–expression coupling (*r* > 0.44). RT-qPCR analysis of 18 candidate genes revealed substantial inter-patient heterogeneity. *NR5A1* exhibited the highest expression variability (coefficient of variation [CV] = 1.486) and significantly higher expression in platinum-responsive tumors than in non-responders (mean 2^⁻ΔCt^ = 0.263 vs. 0.013; *p* < 0.05). Exploratory in silico docking and molecular dynamics (MD) analyses suggested structurally stable binding interactions between Steroidogenic Factor-1 (*SF-1*/*NR5A1*) and the natural plant compound cubebin.

**Conclusion:**

This study demonstrates that histological architecture contains measurable transcriptomic information that can support scalable biomarker prioritization from routine diagnostic histology in HGSOC. NR5A1 represents a hypothesis-generating candidate biomarker and structurally tractable target for future experimental studies. Future validation in larger, multi-center cohorts will be essential to confirm model robustness, biological relevance, and potential clinical utility.

**Supplementary Information:**

The online version contains supplementary material available at 10.1186/s13048-026-02166-y.

## Introduction

 High-grade serous ovarian cancer (HGSOC) is the most aggressive and lethal subtype of ovarian cancer, responsible for approximately 70% of ovarian cancer–related deaths worldwide [1, 2]. Most patients present with advanced-stage disease and widespread peritoneal metastasis. Although primary cytoreductive surgery combined with platinum-based chemotherapy achieves high initial response rates, the majority eventually develop resistance, resulting in relapse and poor long-term survival [3–6]. The extensive molecular and morphological heterogeneity of HGSOC complicates accurate prognostication [7] and therapeutic stratification [8], highlighting the urgent need for robust biomarkers that capture underlying molecular states relevant to treatment response [4].

Transcriptomic profiling has significantly advanced our understanding of HGSOC biology, enabling molecular subtyping, identification of dysregulated pathways, and prediction of treatment sensitivity or resistance [9–11]. Large-scale efforts such as The Cancer Genome Atlas (TCGA) and Clinical Proteomic Tumor Analysis Consortium (CPTAC) have revealed transcriptional programs, immune microenvironmental states, DNA repair deficiencies, and metabolic vulnerabilities relevant to prognosis and therapy [12–14]. However, routine clinical adoption of RNA-sequencing–based assays remains limited by high costs, the need for high-quality fresh or well-preserved tissue, technical complexity, and prolonged turnaround times [15, 16]. Despite their strong mechanistic relevance, transcriptomic biomarkers are therefore not routinely available to guide frontline therapeutic decision-making, particularly in resource-constrained clinical settings [17]. Alternative strategies capable of inferring molecular information without additional tissue sampling are therefore of considerable clinical interest.

Computational pathology has emerged as a promising approach to extract biologically meaningful information directly from routine hematoxylin and eosin (H&E)-stained whole-slide images (WSIs) using machine learning and deep learning methods [18, 19]. Prior studies have demonstrated that histomorphological patterns can encode genomic alterations, molecular subtypes, and clinical outcomes across multiple cancer types [20–23]. However, most existing models focus on prediction of individual genes, predefined molecular features, or pathway-restricted inference, often in pan-cancer cohorts [19–21, 24]. Such strategies may overlook broader transcriptional programs and are less suited to capturing the extensive heterogeneity of HGSOC and remain underexplored, with scarce independent experimental validation.

Recent progress in self-supervised contrastive learning has enabled robust representation learning from large unlabeled histopathology datasets without manual annotation [25, 26]. Contrastive frameworks such as Momentum Contrast (MoCo v2), SimCLR, and DINO encode features related to tumor architecture, stromal organization, nuclear morphology, and inflammatory patterns particularly suited to heterogeneous malignancies like HGSOC [27–31]. These approaches are particularly well suited for complex and heterogeneous diseases, yet their application to genome-wide, multi-output gene expression prediction specifically in HGSOC remains largely unexplored, with limited independent experimental validation.

In this study, we present a virtual transcriptomics framework integrating self-supervised contrastive learning (Momentum Contrast v2 [MoCo v2]) with multi-output Random Forest regression to predict genome-wide expression of approximately 6,400 protein-coding genes directly from H&E whole-slide images of HGSOC. Trained on paired histopathology and RNA-sequencing data from The Cancer Genome Atlas (TCGA), this framework moves beyond single-gene or pathway-restricted inference to enable simultaneous prediction of broad transcriptional programs in a clinically challenging and underrepresented cancer type. To assess biological relevance, predicted transcriptional signatures were experimentally evaluated using reverse transcription quantitative polymerase chain reaction (RT-qPCR) in an independent cohort of treatment HGSOC patients. Candidate genes were prioritized solely based on predictive performance, expression variability, and established roles in ovarian cancer biology. This validation highlighted *NR5A1* (encoding Steroidogenic Factor-1), a transcription factor involved in steroidogenesis and metabolic regulation, as a gene exhibiting pronounced inter-patient variability and association with platinum response. Structure-based molecular docking and molecular dynamics (MD) simulations targeting the SF-1 ligand-binding domain were performed exclusively as proof-of-concept in silico analyses to explore potential druggability, without implying therapeutic efficacy or clinical actionability.

**Fig. 1:**
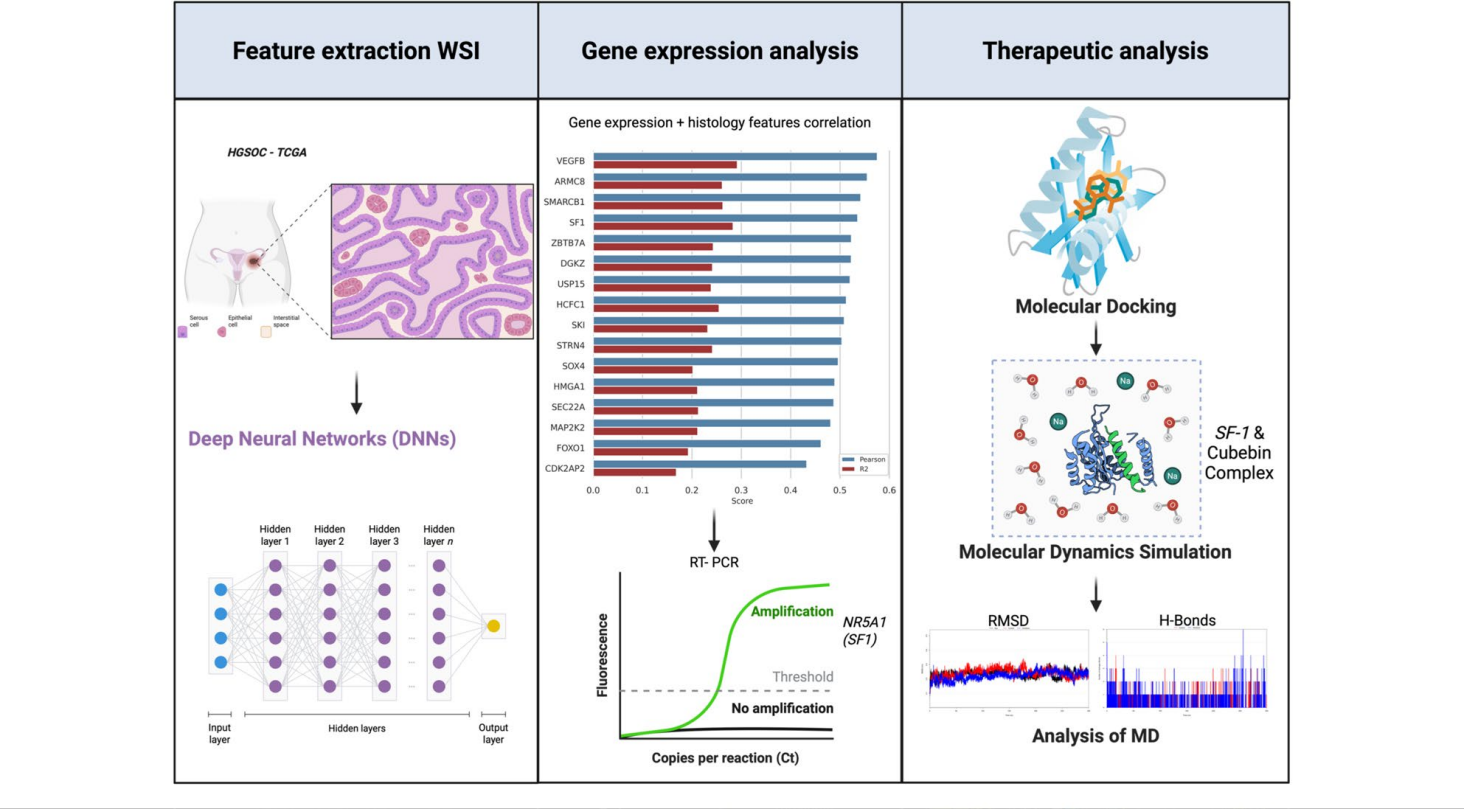
Overview of the integrated computational–experimental framework. Whole-slide H&E images from TCGA high-grade serous ovarian cancer (HGSOC) were processed into tissue-rich tiles and encoded using self-supervised contrastive learning (MoCo v2) to generate slide-level embeddings. These embeddings were used to predict genome-wide gene expression, prioritize candidate genes, and guide RT-qPCR validation in an independent cohort. *NR5A1* emerged as a key candidate and was further evaluated using structure-based molecular docking and molecular dynamics simulations to assess ligand-binding feasibility

Collectively, this study demonstrates that histological architecture contains measurable transcriptomic information that can support biomarker prioritization in HGSOC (Fig. 1). By combining self-supervised image analysis with targeted molecular validation, we establish a scalable, tissue-sparing framework for virtual transcriptomics and data-driven biomarker prioritization from routine diagnostic histology, providing a foundation for future validation and translational development in larger, multi-center cohorts.

## Materials and methods

### Study overview

In this study, we developed a deep learning pipeline to predict genome-wide gene expression profiles directly from H&E WSIs of high-grade serous ovarian cancer (HGSOC) using self-supervised contrastive representation learning and multi-output regression. The workflow comprised: (i) acquisition of paired histopathology WSIs, RNA-sequencing data, and clinical annotations from The Cancer Genome Atlas Ovarian Serous Cystadenocarcinoma (TCGA-OV) cohort; (ii) WSI preprocessing, tissue detection, and tile extraction; (iii) self-supervised representation learning with Momentum Contrast v2 (MoCo v2); (iv) aggregation of tile-level embeddings into slide-level representations; (v) multi-output Random Forest regression to predict transcriptomic profiles from WSIs; and (vi) experimental validation by RT-qPCR in an independent HGSOC cohort, followed by proof-of-concept structure-based molecular docking and dynamics simulations for druggability analysis.

### TCGA-HGSOC cohort acquisition and data selection

A total of 1,371 diagnostic H&E WSIs were retrieved, comprising 1,208 tumor WSIs and 163 normal or adjacent non-tumor WSIs. RNA-sequencing (RNA-seq) data and clinical annotations for HGSOC were obtained from the TCGA-OV project via the Genomic Data Commons (GDC) data portal (https://portal.gdc.cancer.gov/ ; accessed 2 January 2024) [9, 32, 33]. All files were downloaded using the GDC Data Transfer Tool in accordance with TCGA publication guidelines. Cases were included if they had diagnostic H&E slides of primary ovarian tumors, matched RNA-seq expression data (HTSeq-FPKM-UQ), and confirmed high-grade serous histology. Slides with inadequate staining, major artifacts, or missing matched RNA-seq data were excluded. For patients with multiple slides, a single representative slide was selected based on tumor content and staining quality. Normal and adjacent non-tumor slides were excluded from model training and used only for quality control. All TCGA data were publicly available and de-identified; therefore, no additional ethical approval was required. RNA-seq values were log_2_-transformed [log_2_(FPKM-UQ + 1)]. Non-protein-coding genes, zero-variance genes, and those missing in > 5% of samples were removed, yielding ~ 6,400 protein-coding targets after Ensembl v98 harmonization. Clinical metadata (age, FIGO stage, debulking status, platinum response, survival) were extracted. Additional cohort selection and preprocessing details are provided in Supplementary Methods S1.

### WSI preprocessing and tile extraction

WSIs were processed using the OpenSlide library [34]. Tissue regions were identified by generating a tissue mask on downsampled images using color-based thresholding (HSV Otsu) and morphological filtering [35]. No color normalization was applied to preserve native staining variability. Tiles of size 256 × 256 pixels were extracted at an effective 20× magnification from tissue-rich regions only, requiring ≥ 60% tissue overlap. Tiles were filtered for sharpness, intensity, and absence of artifacts or pen marks. Up to 10,000 tiles per WSI were randomly sampled to balance representation. Patient-level partitioning prevented data leakage across folds. No stain normalization was applied; variability was addressed through augmentations. Full preprocessing parameters, tissue masking thresholds, artifact filtering criteria, and tile subsampling details are provided in Supplementary Methods S2.

### Self-supervised representation learning

MoCo v2 contrastive learning was used to extract morphological features from H&E tiles without manual annotations [26]. ResNet-50 was selected due to its widespread use and proven performance in histopathology representation learning. The framework builds on the original Momentum Contrast formulation [36] and contrastive representation learning with InfoNCE [37]. Tile-level representations were learned using a ResNet-50 [38] backbone trained for 200 epochs on augmented tile pairs, maximizing agreement between positive pairs while contrasting negatives stored in a dynamic queue. Data augmentations included color jittering, Gaussian blur, geometric transformations, and stain-preserving perturbations to promote invariance to staining and scanner variability. After training, the ResNet-50 encoder was used to extract 1,024-dimensional tile-level feature embeddings for downstream analyses. Full architectural details and training hyperparameters are provided in Supplementary Methods S3 and Supplementary Table S2.

### Slide-level embedding construction

After self-supervised pretraining, tile-level embeddings were aggregated into fixed-dimensional slide-level representations to enable transcriptome prediction. For each WSI, embeddings from valid tiles were summarized using permutation-invariant statistical pooling (mean, standard deviation, minimum, and maximum), yielding a 4,096-dimensional feature vector per slide [39, 40]. This permutation-invariant approach captures both dominant and rare morphological patterns while accommodating variable tile counts across slides. The exact mathematical formulation of tile-to-slide pooling and embedding reproducibility settings are provided in Supplementary Methods S4.

### Multi-output regression for transcriptome prediction

Gene expression prediction was formulated as a multi-output regression task using a Random Forest model (500 trees; multi-output mode enabled) [41, 42]. Random Forests were chosen for interpretability and robustness; future work may explore neural regressors. Slide-level embeddings served as input features, and matched log₂-transformed RNA-seq expression values for approximately 6,400 protein-coding genes were used as outputs. Model performance was assessed using five-fold patient-level cross-validation, with prediction accuracy quantified using Pearson correlation between predicted and observed expression. Full model configuration and evaluation settings are detailed in Supplementary Methods S5 and Supplementary Table S3.

To clarify dataset splitting and leakage prevention, model training and evaluation were performed using five-fold patient-level cross-validation. All WSIs and all tiles belonging to the same patient were assigned to a single fold, ensuring that no patient contributed data to both training and validation sets. For downstream transcriptome prediction, slide-level embeddings were used to train the multi-output Random Forest regressor on the training folds, and performance was evaluated on held-out patient-level validation folds. To reduce overfitting, tile sampling was capped at 10,000 tissue-rich tiles per WSI, low-quality and artifact-containing tiles were excluded, stain and geometric augmentations were applied during contrastive learning, and Random Forest hyperparameters were fixed before cross-validation. Detailed model settings are provided in Supplementary Methods S5 and Supplementary Table S3.

### RT-qPCR validation cohort and gene expression analysis

#### Patient cohort and sample collection

Ten HGSOC tissue samples were obtained between 2021 and 2024 at the University Clinical Hospital No. 1 in Lublin, Poland, from women aged 51–80 years. This cohort size was selected based on sample availability to provide an initial proof-of-concept validation of our computational predictions. All tumors were classified as FIGO stage III and histological grade G2–G3, as assessed by experienced pathologists. Tumor tissue samples were collected intraoperatively during primary debulking surgeries prior to the initiation of any oncological treatment and were preserved in RNAlater Stabilization Solution (Thermo Fisher Scientific, Waltham, MA, USA) and stored at − 80 °C until further analysis. Following surgery, patients received adjuvant doublet chemotherapy consisting of six cycles of carboplatin (AUC6) and paclitaxel (175 mg/m^2^). Inclusion criteria comprised histologically confirmed HGSOC, no previous anticancer therapy, and age ≥ 18 years. All participants provided written informed consent. The study was approved by the Bioethical Committee of the Medical University of Lublin (KE-0254/20/2021, KE-0254/185/10/2022, and KE-0254/23/01/2024).

#### Clinical classification

Clinical response to chemotherapy was evaluated using contrast-enhanced CT scans performed 6 months after completion of treatment. Tumor response was assessed by an experienced radiologist using Response Evaluation Criteria In Solid Tumors (RECIST 1.1) criteria, blinded to all other clinical and molecular data. Patients were classified as responders (complete response [CR], partial response [PR], or no evidence of disease [NED]; coded as 1) or non-responders (progressive disease [PD]; coded as 0). In total, 4 patients were classified as responders and 6 as non-responders. Clinicopathological characteristics and response status are summarized in (Table 1).

**Table 1:** Clinicopathological characteristics and treatment response of patients with high-grade serous ovarian cancer (HGSOC)

Patients	FIGO	GRADE	Response
HGSOC_01	IIIC	G3	0
HGSOC_02	IIIC	G3	0
HGSOC_03	IIIB	G2	1
HGSOC_04	IIIC	G3	1
HGSOC_05	IIIB	G2	0
HGSOC_06	IIIC	G2	0
HGSOC_07	IIIB	G2	0
HGSOC_08	IIIC	G2	0
HGSOC_09	III	G3	1
HGSOC_10	II/III	G2	1

FIGO and histological grading were assessed by experienced pathologists. Response was coded as 0 = non-responder (PD), 1 = responder (CR, PR, or NED). *CR* Complete response, *PR* Partial response, *PD* Progressive disease, *NED* No evidence of disease

#### RT-qPCR gene expression analysis

RNA was extracted using TRIzol [43] and cDNA was synthesized from 1 µg total RNA. RT-qPCR used SYBR Green on Applied Biosystems 7500 Fast in triplicates. Eighteen candidate genes were profiled: *VEGFB*,* SMARCB1* (SNF5), *NR5A1*,* SKI*,* MAP2K2 (MEK2)*,* ZBTB7A*,* DGKZ*,* USP15*,* MLLT1*,* ARMC8*,* HCFC1*,* STRN4*,* SEC22L2*,* HMGA1*,* SOX4*,* FOXO1*,* CDK2AP2*, and the reference genes *ACTB* and *RNA18SN5*. Expression was calculated using the 2⁻^ΔCt^ method with geometric mean normalization to *ACTB* and *RNA18SN5* [44], following MIQE reporting recommendations [45]. Candidate genes were prioritized for validation based on predictive performance, expression variability, and biological relevance. Primer sequences and assay quality-control criteria are provided in Supplementary Methods S6 and Supplementary Table S1.

### Structure-based molecular docking

Structure-based molecular docking was performed as a proof-of-concept analysis to explore the structural druggability of SF-1 without implying therapeutic efficacy. The crystallographic structure of the SF-1 ligand-binding domain (PDB ID: 4QJR) was retrieved from the Protein Data Bank. Virtual screening of a curated small-molecule library (Supplementary Table S4) was conducted using AutoDock Vina [46]. Docking was focused on the predicted ligand-binding pocket of SF-1, and binding affinities were used to rank candidate compounds. Reference compounds with previously reported SF-1-related activity were included to benchmark docking plausibility. Candidate selection criteria and full docking protocols, pocket identification procedures, and compound details are provided in Supplementary Methods S7.

### Molecular dynamics simulations and binding free energy calculations

Molecular dynamics (MD) simulations were conducted to assess the structural stability of SF-1 in apo form and in complex with selected ligands, exclusively as an in silico feasibility analysis. Simulations were performed using GROMACS (version 2023.2) with the CHARMM36 force field for the protein and CGenFF for ligands [47, 48]. Each system was simulated for 300 ns following standard equilibration procedures. Systems were minimized and equilibrated (NVT/NPT), analyzed for root mean square deviation (RMSD) /root mean square fluctuation (RMSF) /radius of gyration (Rg) /hydrogen bonds [49, 50]. Trajectories were analyzed to evaluate conformational stability and protein–ligand interactions. Binding free energies were estimated using MM/PBSA and MM/GBSA approaches [51]. Detailed simulation parameters, system preparation steps, and analysis workflows are described in Supplementary Methods S8.

### Statistical analysis

Model performance was evaluated using Pearson correlation coefficients between predicted and observed RNA-sequencing expression values in held-out patient-level folds. RT-qPCR group comparisons were performed using unpaired two-tailed t-tests. No multiple-testing correction was applied to RT-qPCR analyses due to the exploratory nature of the validation. Given the exploratory nature of this validation and limited cohort size, results are interpreted as hypothesis-generating, and all response-associated findings require validation in larger independent cohorts. All analyses were conducted using Python and R. Predicted and observed gene-expression matrices used for performance evaluation are provided as Supplementary Data S1–S2.

## Results

### Deep learning framework for image-based virtual transcriptome prediction

**Fig. 2:**
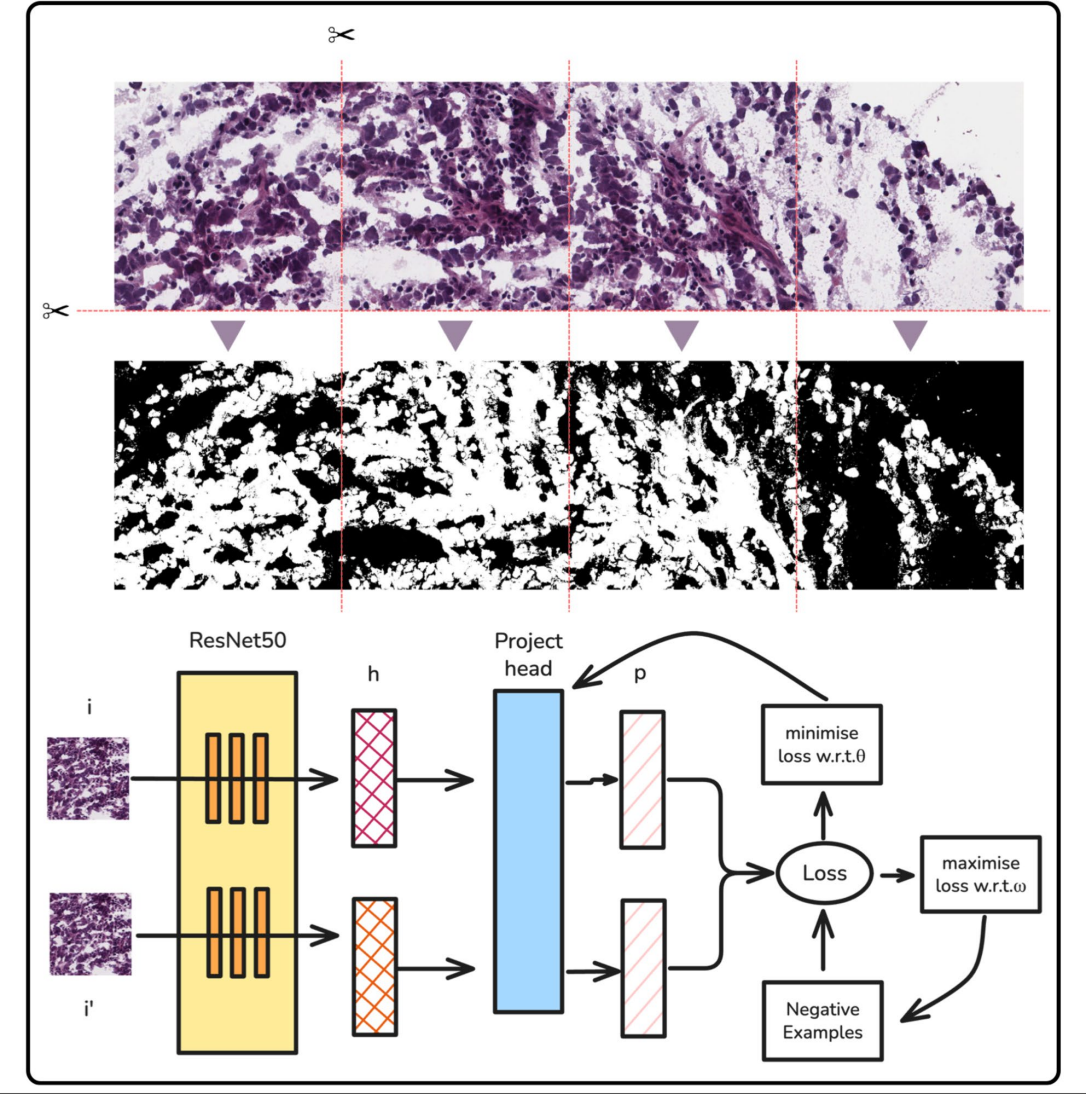
Overview of WSI preprocessing and self-supervised contrastive representation learning. **A** Representative example of tissue-mask generation and tile extraction from H&E whole-slide images. Upper panels show original image tiles; lower panels depict corresponding binary tissue masks used to restrict analysis to tissue-rich regions. **B** Schematic of the Momentum Contrast v2 (MoCo v2) self-supervised learning framework with a ResNet-50 backbone. Augmented tile pairs (positive pairs) are encoded into feature embeddings and optimized using a contrastive objective that maximizes similarity between positive pairs while contrasting against a large set of negative examples

To evaluate whether histological information captured in routine hematoxylin and eosin (H&E)–stained whole-slide images (WSIs) can be leveraged to infer transcriptional states in high-grade serous ovarian cancer (HGSOC), we developed a deep learning–based virtual transcriptomics framework (Fig. 2). The framework integrates automated WSI preprocessing, self-supervised representation learning, and multi-output regression to enable genome-wide gene expression prediction directly from histopathology images. WSIs from the TCGA-OV cohort were first subjected to automated tissue detection to identify tissue-rich regions while excluding background glass and artifacts. Resulting in 5,000–30,000 tiles per slide, from which up to 10,000 tiles were sampled per WSI for embedding generation. Representative examples of tissue-mask generation and tile extraction are shown in Fig. 2A. From each slide, fixed-size image tiles were extracted at 20× magnification following quality filtering, ensuring consistent input for downstream analysis across heterogeneous samples.

To avoid reliance on manual annotations or predefined histological features, tile-level representations were learned using self-supervised contrastive learning based on the Momentum Contrast v2 (MoCo v2) framework. In this approach, two augmented views of each tile were processed through a shared ResNet-50 encoder, and the resulting representations were optimized to maximize agreement between positive pairs while maintaining separation from a large set of negative examples stored in a dynamic memory queue (Fig. 2B). This resulted in 1,024-dimensional tile embeddings capturing salient morphological patterns, including nuclear pleomorphism, stromal architecture, and tumor–stroma interface structure. This training strategy enables the model to learn robust and invariant morphological features directly from H&E images without access to clinical or molecular labels.

Following self-supervised pretraining, tile-level embeddings were aggregated into fixed-length slide-level representations using permutation-invariant statistical pooling. These slide-level features served as inputs for a multi-output regression model trained to predict genome-wide gene expression profiles. Model training and evaluation were performed using patient-level cross-validation to prevent information leakage between training and validation sets. This study design establishes an end-to-end framework for linking histological architecture to transcriptional programs in HGSOC and provides the foundation for subsequent evaluation of genome-wide prediction performance, biological prioritization of candidate genes, and independent experimental validation.

### Genome-wide prediction of gene expression from H&E whole-slide images

We next evaluated whether slide-level representations derived from self-supervised contrastive learning could predict genome-wide gene expression profiles in the TCGA-OV HGSOC cohort. Fixed-length slide-level embeddings were constructed from tile-level features and used to train a multi-output Random Forest regression model to infer RNA-sequencing expression values for approximately 6,400 protein-coding genes. Across all predicted genes, the model achieved a mean Pearson correlation of *r* = 0.36 between predicted and observed expression values, indicating that a substantial fraction of transcriptomic variation in HGSOC is encoded within routine histopathological images. This genome-wide average reflects performance across the full set of approximately 6,400 protein-coding genes, including many genes with weak or no morphological imprint. Prediction performance was consistent across five-fold patient-level cross-validation, with no overlap of patients between training and validation folds, supporting the robustness and generalizability of the learned representations within the TCGA cohort. These correlations indicate that histology-derived features capture measurable transcriptomic signal, but the observed performance should be interpreted as supporting biomarker prioritization rather than individual-level clinical decision-making.

**Fig. 3:**
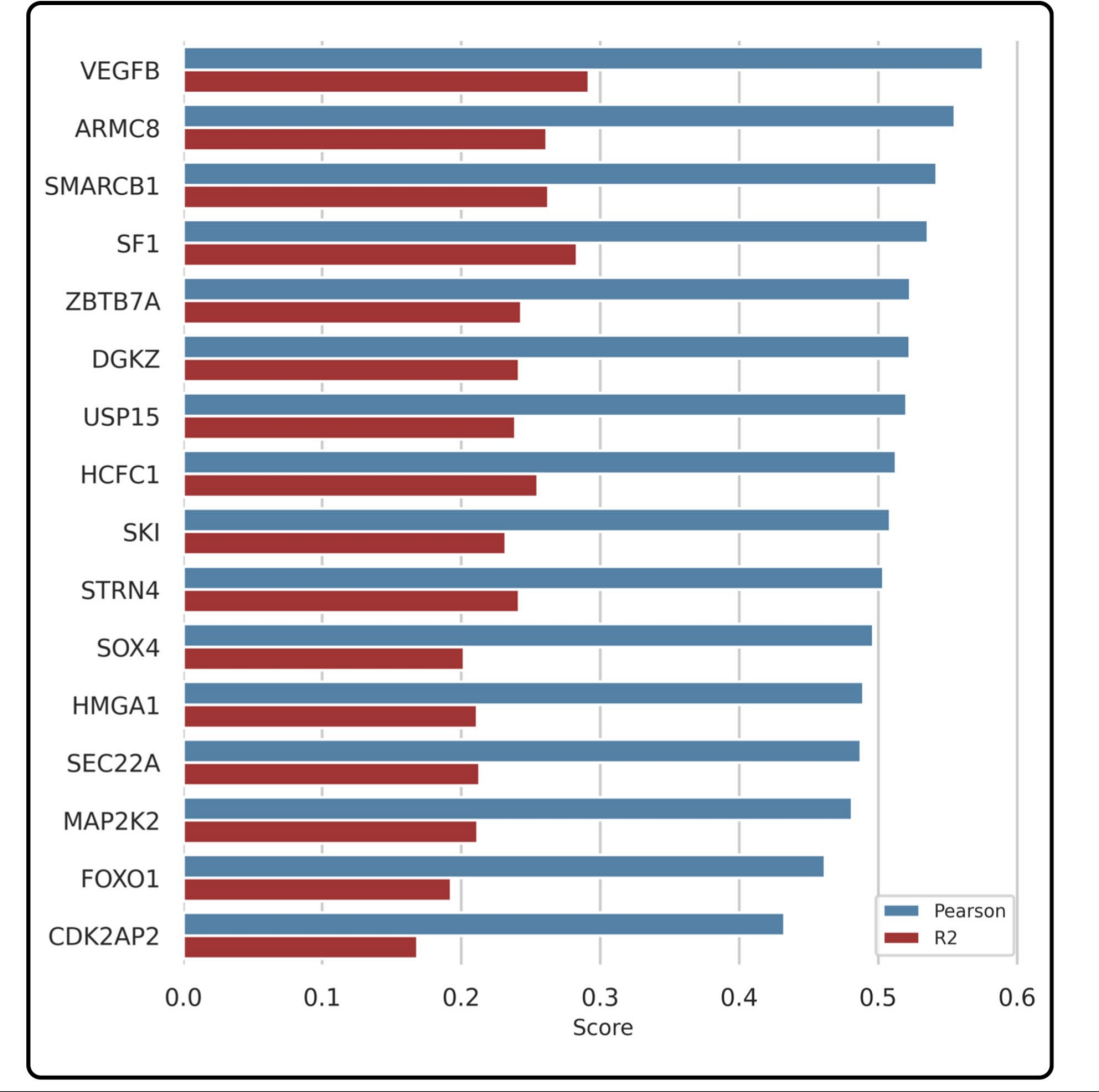
Gene-wise prediction performance for transcriptome inference from H&E whole-slide images. Bar plots showing Pearson correlation coefficients (blue) and coefficients of determination (R²; red) for the top 18 genes ranked by cross-validated prediction performance in the TCGA HGSOC cohort

Gene-wise prediction performance was heterogeneous. While many genes exhibited low predictability (near-zero correlation), consistent with limited morphological correlates or dominant contributions from non-tumor cell populations, a distinct subset demonstrated stronger and reproducible associations between histological features and gene expression. Approximately 300 genes achieved Pearson correlations exceeding *r* > 0.44, representing a higher-confidence group with stronger structure–expression coupling for biologically relevant transcriptional programs (Fig. 3) displaying Pearson correlation coefficients alongside coefficients of determination (R²). Highly predictable genes included *VEGFB, ARMC8, SMARCB1, NR5A1, ZBTB7A, USP15, HCFC1, DGKZ*, and other regulators involved in transcriptional control, signaling, and tumor–microenvironment interactions. The prominence of transcription factors, signaling mediators, and chromatin-associated genes among top performers is consistent with prior observations that histology-based models preferentially capture proliferative, stromal, and microenvironmental features reflected in gene expression profiles.

**Fig. 4:**
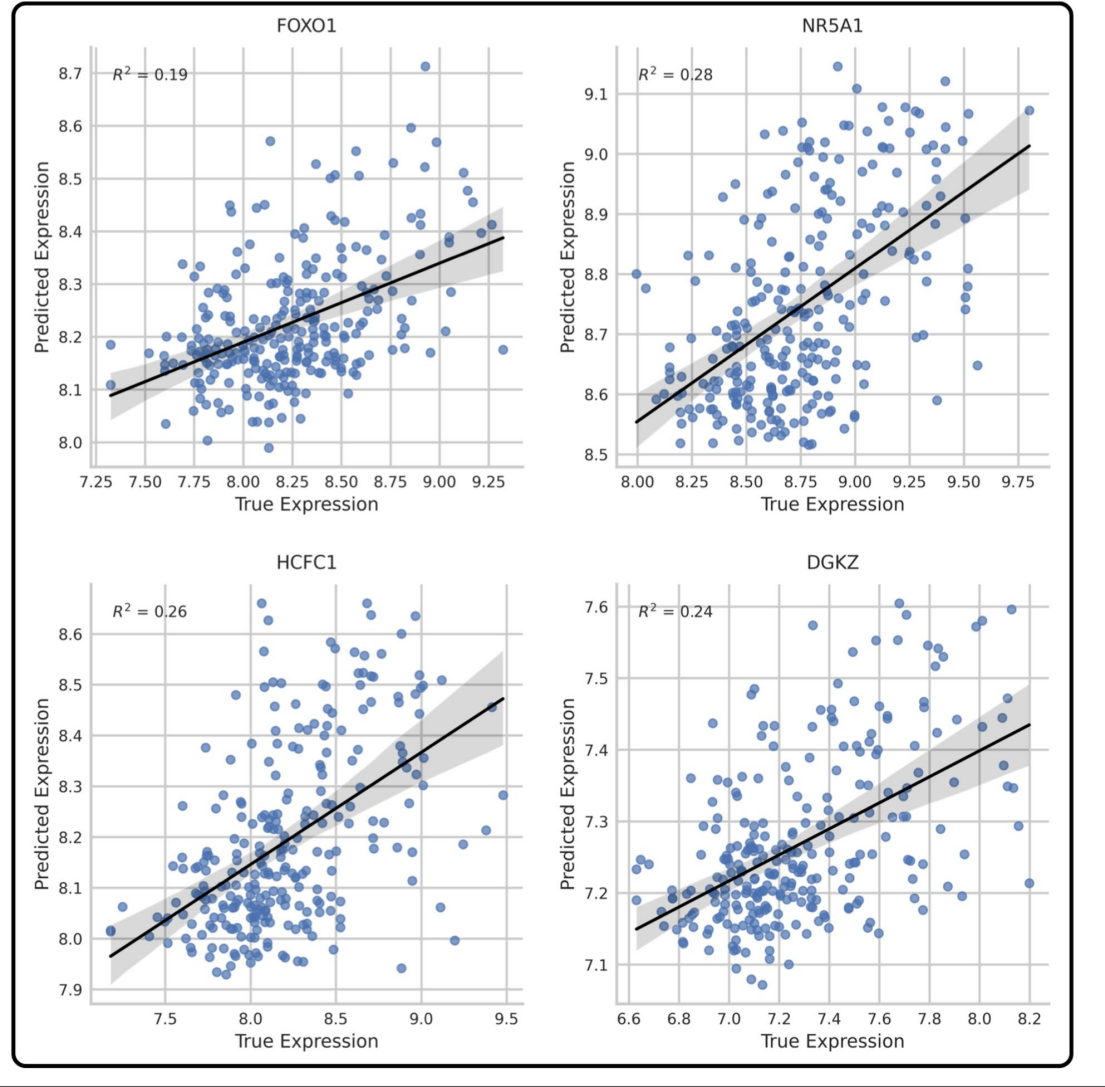
Predicted versus observed gene expression for representative genes. Scatterplots comparing predicted and RNA-sequencing–derived expression values for representative genes (*FOXO1, NR5A1, HCFC1*, and *DGKZ*) across TCGA HGSOC samples. Solid lines indicate linear regression fits; shaded regions denote 95% confidence intervals. The reproducible linear associations support prioritization of highly predictable genes for experimental validation

To further illustrate gene-specific prediction behavior, Fig. 4 presents scatterplots comparing predicted and observed expression values for representative genes. *NR5A1* demonstrated among the strongest linear associations (R² ≈ 0.28), whereas *FOXO1* showed moderate predictability (R² ≈ 0.19). Additional examples, including *HCFC1* and *DGKZ*, further highlight consistent gene-specific patterns across the cohort. These relationships indicate that the model captures reproducible, gene-dependent associations between histological architecture and transcript abundance. Analysis of feature importance across Random Forest models revealed that multiple embedding dimensions consistently contributed to gene-expression prediction, suggesting that the learned representations encode stable morphological information relevant to transcriptional variation. While direct biological interpretation of individual embedding dimensions remains limited, these findings support the notion that contrastive learning captures composite histological patterns that reflect underlying molecular programs in HGSOC.

Taken together, these results demonstrate that self-supervised representation learning coupled with multi-output regression enables scalable, genome-wide inference of gene expression from routine H&E-stained WSIs.

The observed performance compares favorably with previously reported histology-based transcriptome prediction frameworks applied across heterogeneous cancer types [21], highlighting the benefit of disease-specific modeling and tailored self-supervised pretraining in HGSOC.

### Prioritization of candidate genes from image–transcriptome predictions

To identify genes for orthogonal experimental evaluation, we first ranked all predicted genes by cross-validated image–transcriptome performance in the TCGA-HGSOC cohort (Pearson correlation between predicted and observed RNA-seq), prioritizing genes with consistent performance across folds and plausible relevance to HGSOC biology (e.g., angiogenesis, chromatin regulation, signaling, transcriptional control, and stress-response pathways). This image-based ranking yielded a subset of genes with strong structure–expression coupling, including regulators of angiogenesis (e.g., *VEGFB*), chromatin remodeling and transcriptional programs (e.g., *SMARCB1/INI1*,* MLLT1*,* WDR5B*,* HCFC1*), MAPK signaling (*MAP2K2(MEK2*)), and ubiquitin-related mechanisms (*USP15*,* ARMC8*). From this ranked list, we assembled an 18-gene RT-qPCR validation panel designed to span functional diversity while maintaining high image-based predictability. The panel included angiogenesis (*VEGFB*), chromatin remodeling/transcriptional regulation (*SMARCB1*,* MLLT1*,* WDR5B*,* HCFC1*,* HMGA1*,* ZBTB7A*,* SKI*,* FOXO1*,* SOX4*), signaling and lipid metabolism (*MAP2K2*,* DGKZ*), ubiquitin/deubiquitination (*ARMC8*,* USP15*), vesicle trafficking (*SEC22L2*), and cell-cycle associated regulation (*CDK2AP2*), together with the nuclear receptor SF-1 (*NR5A1*). This strategy ensured that the validation set did not overrepresent a single pathway and better reflected the multi-program nature of HGSOC.

To provide interpretability for the shortlisted candidates, we annotated high-performing genes by functional category and potential clinical relevance (Supplementary Table 5). For example, VEGFB represents a key angiogenesis-associated factor; angiogenic programs are therapeutically actionable in HGSOC, where anti-angiogenic strategies have established clinical relevance [52]. Genes involved in chromatin remodeling and transcriptional control (e.g., *SMARCB1/INI1*,* MLLT1*,* WDR5B*,* HCFC1*) are also notable because epigenetic dysregulation is a recurring feature of HGSOC and may shape morphology-linked transcriptional states. At this stage, *NR5A1* was included in the RT-qPCR validation panel because it combined strong image-based predictability with biological relevance to ovarian steroidogenic and metabolic programs. Its subsequent prioritization for structural analyses was based on convergence of image-derived predictability, RT-qPCR variability, response-associated expression, biological plausibility, and availability of a resolved ligand-binding domain, rather than on a single metric.

### RT-qPCR validation in an independent HGSOC cohort

To experimentally assess the computationally inferred gene-expression signatures, RT-qPCR was performed on tumor samples from an independent cohort of ten FIGO stage III high-grade serous ovarian cancers (HGSOC), stratified into platinum responders (*n* = 4) and non-responders (*n* = 6) according to RECIST 1.1 criteria. Expression of 18 candidate genes (*VEGFB*,* SMARCB1*,* NR5A1*,* SKI*,* MAP2K2*,* ZBTB7A*,* DGKZ*,* USP15*,* MLLT1*,* ARMC8*,* WDR5B*,* HCFC1*,* STRN4*,* SEC22L2*,* HMGA1*,* SOX4*,* FOXO1*, and *CDK2AP2*) was quantified and normalized to the geometric mean of two reference genes (*ACTB* and *RNA18SN5*) using the 2^⁻ΔCt^ method. Across the 18 targets, substantial inter-patient expression heterogeneity was observed. The coefficient of variation (CV) of normalized 2^⁻ΔCt^ values ranged from 0.215 (SKI) to 1.486 (*NR5A1*), indicating marked differences in expression stability between genes. *NR5A1* displayed the highest variability, followed by *SOX4* (CV = 0.758), *HMGA1* (CV = 0.746), *CDK2AP2* (CV = 0.697), *VEGFB* (CV = 0.447), and *SMARCB1* (CV = 0.332) (Table 2). This pronounced heterogeneity suggests that *NR5A1* expression is highly context-dependent in HGSOC, potentially reflecting differences in tumor-intrinsic programs and microenvironmental influences.

**Table 2:** Coefficient of variation (CV) of RT-qPCR expression (2⁻ΔCt) across 10 HGSOC samples

Gene	CV	Gene	CV	Gene	CV
*VEGFB*	0.447	*SMARCB1*	0.332	*NR5A1*	1.486
*SKI*	0.215	*MAP2K2*	0.549	*ZBTB7A*	0.321
*DGKZ*	0.404	*USP15*	0.400	*MLLT1*	0.431
*ARMC8*	0.411	*WDR5B*	0.415	*HCFC1*	0.411
*STRN4*	0.415	*SEC22L2*	0.410	*HMGA1*	0.746
*SOX4*	0.758	*FOXO1*	0.642	*CDK2AP2*	0.697

When stratified by treatment response, several genes showed numerically higher expression in platinum-responsive tumors, although statistical power was limited by cohort size. Among all candidates, *NR5A1* demonstrated the most pronounced response-associated difference, with approximately five-fold higher mean expression in responders (mean 2⁻ΔCt = 0.263; Ct ≈ 21.93) compared with non-responders (mean = 0.013; Ct ≈ 26.27). This difference reached nominal statistical significance (*p* < 0.05, unpaired two-tailed t-test) and was associated with a large effect size (Cohen’s d), supporting a potentially biologically meaningful separation despite the small sample size. Because no multiple-testing correction was applied to this exploratory screen, this result should be interpreted as hypothesis-generating. Other genes, including *SMARCB1*,* HMGA1*, and *SOX4*, showed trends toward higher expression in responders but with more modest effect sizes and without statistical significance. *SMARCB1* additionally exhibited grade-associated variation, with higher expression in grade G2 compared with G3 tumors, although this trend did not reach statistical significance. *CDK2AP2* showed minimal differences between responders and non-responders.

**Fig. 5:**
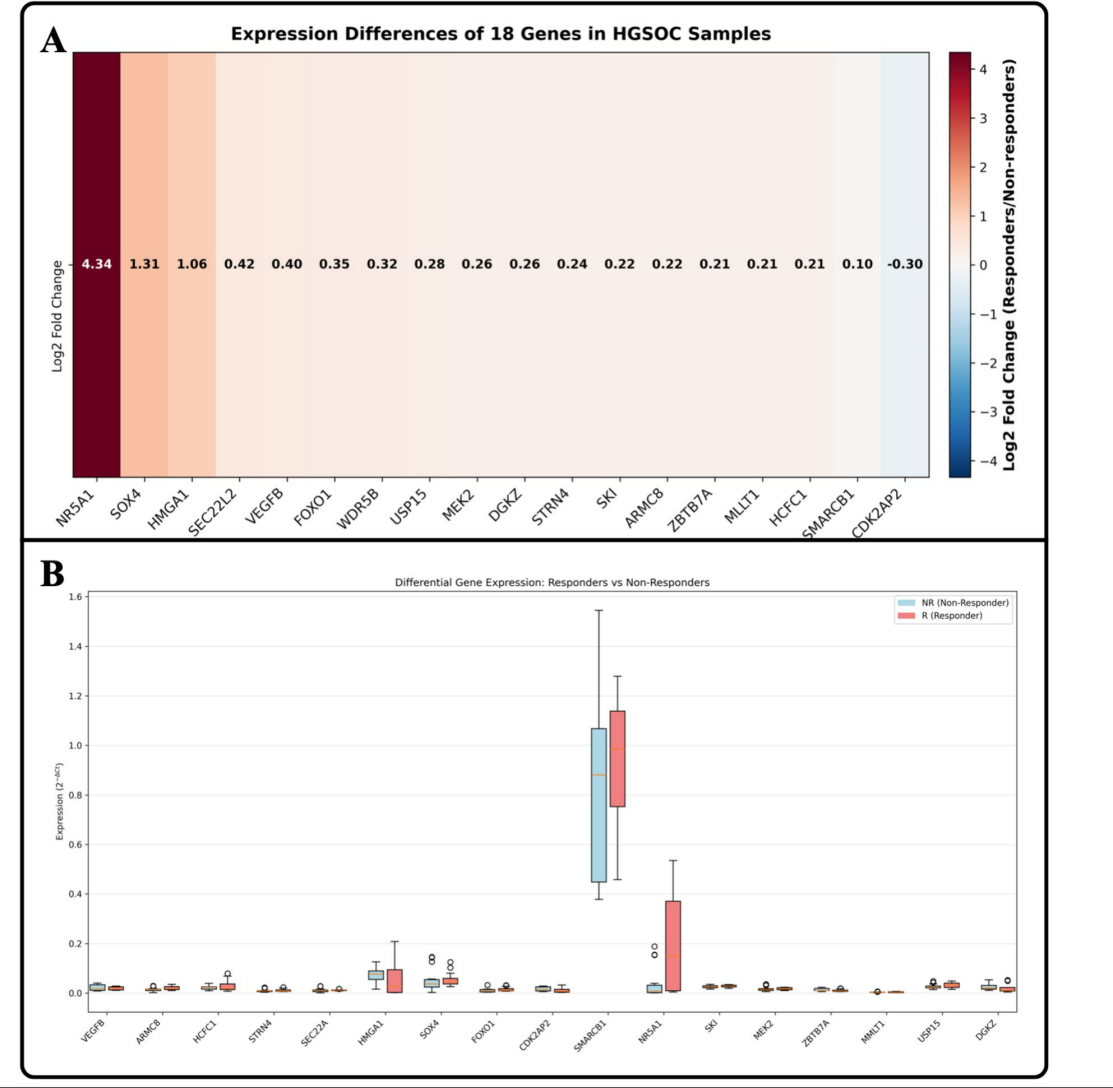
Differential expression of candidate genes in high-grade serous ovarian cancer (HGSOC) responders and non-responders measured by RT-qPCR. **A** Heatmap showing log₂ fold changes in relative mRNA expression for 18 genes between responders and non-responders based on RT-qPCR analysis. Color intensity represents the magnitude and direction of differential expression, and values indicate log₂(responder/non-responder) fold changes. **B** Boxplots comparing normalized RT-qPCR expression levels in responders (R, red) and non-responders (NR, blue). Boxes represent interquartile ranges, center lines indicate medians, whiskers show variability, and points denote outliers

To visualize global expression patterns, log₂ fold changes between responders and non-responders were calculated for all genes. To distinguish group-level expression differences from inter-patient variability, response-associated changes were visualized both as log₂ fold changes (Fig. 5A) and as absolute normalized expression distributions across individual tumors (Fig. 5B). As shown in Fig. 5A, *NR5A1* exhibited the largest relative upregulation (log₂ fold change ≈ 4.34). Figure 5B presents boxplots for selected genes with high variability or biological relevance, highlighting *NR5A1* as the only candidate showing a clear and statistically supported separation between response groups. Collectively, these findings identify *NR5A1* as the most robustly differentiated gene in the validation cohort and support its prioritization for subsequent proof-of-concept structural druggability analyses, while underscoring the need for validation in larger, independent cohorts.

### Integration of image-based predictions with RT-qPCR findings

Finally, we integrated image-based transcriptome predictions from the TCGA HGSOC cohort with RT-qPCR measurements obtained from the independent validation cohort. Genes that were both robustly predictable from whole-slide images and exhibited high inter-patient variability by RT-qPCR were considered the most promising candidates for downstream translational investigation. Among the profiled genes, *NR5A1* satisfied both criteria, showing relatively strong image-based prediction performance in the MoCo v2–Random Forest framework and the highest expression variability in the RT-qPCR cohort, together with a consistent trend toward higher expression in platinum-responsive tumors. Other genes in the validation panel, including *VEGFB*,* HMGA1*,* SOX4*, and *FOXO1*, demonstrated intermediate variability and more modest associations with treatment response, suggesting that they may contribute to composite multi-gene signatures rather than acting as single-gene markers.

Collectively, these findings demonstrate the feasibility of integrating contrastive-learning–derived histological representations with molecular validation to prioritize transcriptional candidates from routine diagnostic histology. This integrated approach highlights *NR5A1* as a biologically and clinically relevant candidate emerging from image-based transcriptome inference and supports its further evaluation in larger, clinically annotated HGSOC cohorts. Based on these findings, *NR5A1* was selected for proof-of-concept structure-based docking and molecular dynamics analyses.

### Structure-based modeling identifies *NR5A1* as a structurally tractable candidate target

*NR5A1* was prioritized for downstream structure-based analysis because it satisfied multiple independent selection criteria. First, *NR5A1* belonged to the subset of genes with stronger image-based predictability in the TCGA-HGSOC cohort, supporting structure–expression coupling between histological architecture and *NR5A1* expression. Second, in the independent RT-qPCR cohort, *NR5A1* showed the highest inter-patient expression variability among all 18 candidate genes, with a coefficient of variation of 1.486. Third, *NR5A1* showed the clearest response-associated expression difference, with higher expression in platinum-responsive tumors than in non-responders. Fourth, *NR5A1* encodes Steroidogenic Factor-1, a nuclear receptor involved in steroidogenic, metabolic, and differentiation-related programs relevant to ovarian biology. Finally, the availability of a resolved ligand-binding domain structure provided a rational basis for exploratory docking and molecular dynamics analyses. Thus, *NR5A1* was selected not on the basis of a single metric, but through convergence of image-derived predictability, molecular validation, biological plausibility, and structural tractability. Based on this convergent prioritization, *NR5A1/SF-1* was selected for proof-of-concept in silico ligand screening and molecular dynamics simulations using the resolved SF-1 ligand-binding domain structure (PDB ID: 4QJR).

### Molecular docking reveals high-affinity ligands for the SF-1 ligand-binding domain

**Fig. 6:**
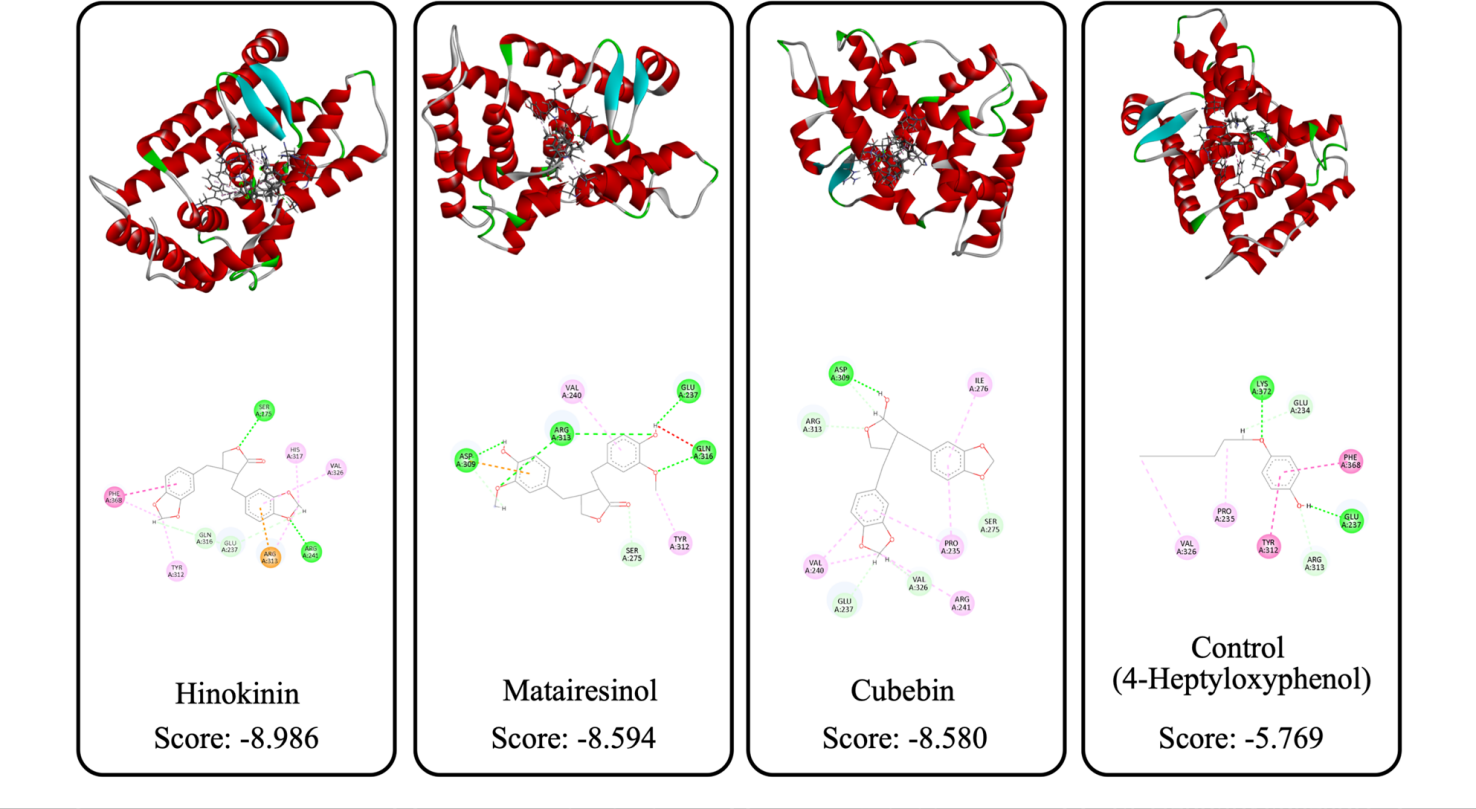
Docking poses and interaction profiles of the top ligands bound to the *NR5A1* ligand-binding domain (4QJR). For each ligand, the upper panel shows the predicted three-dimensional binding pose, while the lower panel depicts the corresponding two-dimensional protein–ligand interaction map

Virtual screening against the SF-1 ligand-binding pocket identified three natural compounds hinokinin, matairesinol, and cubebin with the most favorable predicted binding affinities (Fig. 6). Hinokinin exhibited the strongest docking score (− 8.99 kcal·mol⁻¹), forming multiple hydrophobic interactions and two hydrogen bonds within the pocket. Matairesinol showed a comparable binding score (− 8.59 kcal·mol⁻¹) and engaged in an extensive hydrogen-bonding network, although one unfavorable donor–donor interaction was observed. Cubebin displayed a similarly favorable docking score (− 8.58 kcal·mol⁻¹), dominated by hydrophobic and π–alkyl contacts. For benchmarking, the known SF-1 antagonist 4-(heptyloxy)phenol was included as a control and showed weaker predicted binding affinity and less extensive interaction profiles, consistent with prior biochemical reports. Based on docking scores, interaction geometry, and pocket occupancy, these four ligands were selected for MD simulations.

### Molecular dynamics simulations reveal differential stability of SF-1–ligand complexes

**Fig. 7:**
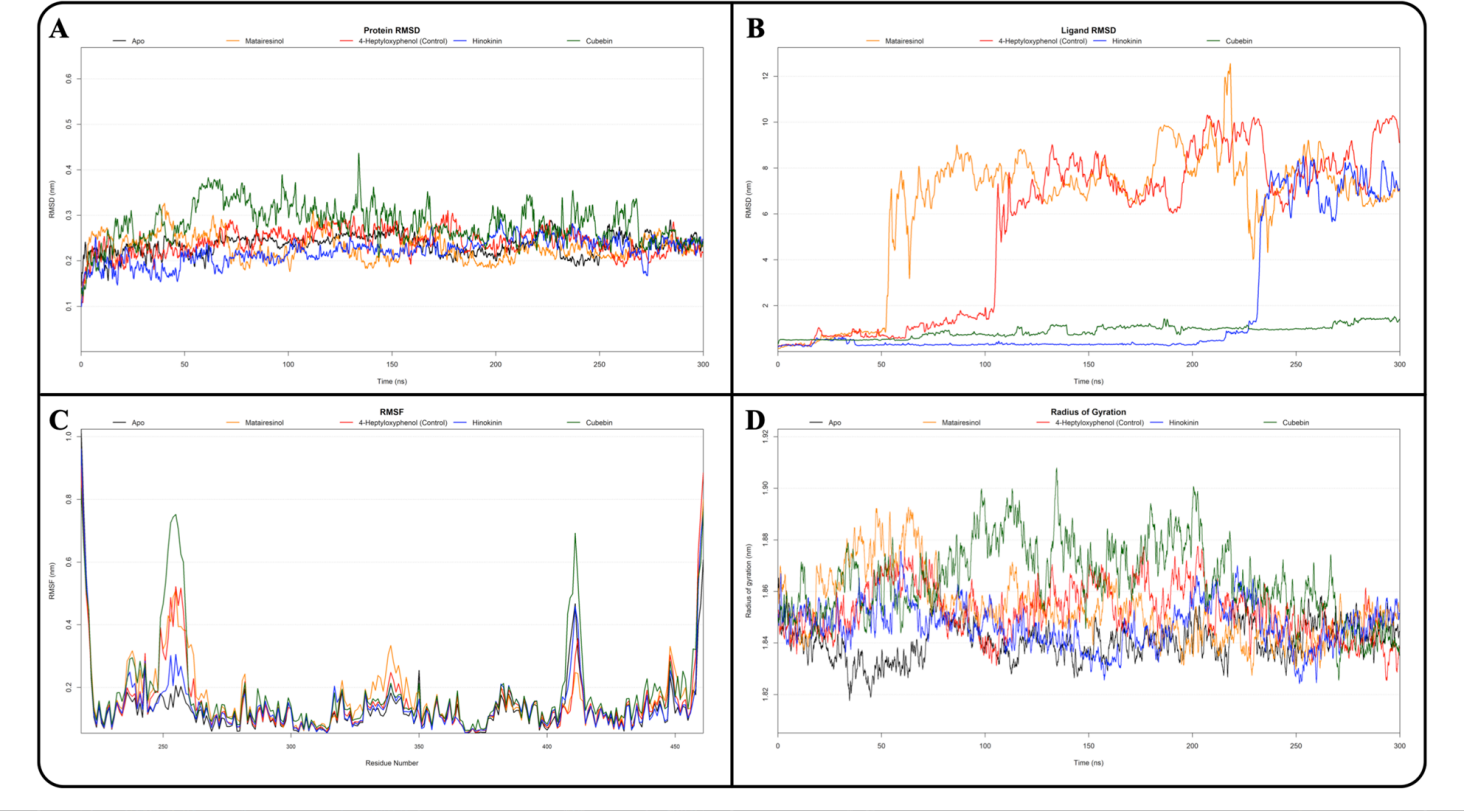
Structural stability and flexibility analysis of the apo 4QJR protein and its ligand-bound complexes during molecular dynamics simulations. (**A)** Protein backbone RMSD (**B**) Ligand heavy-atom RMSD (**C**) RMSF of the protein residues (**D**) Radius of gyration (Rg) of the protein

To assess dynamic stability, 300-ns MD simulations were performed for apo SF-1 and all ligand-bound complexes. Protein backbone RMSD values converged rapidly and remained stable across all systems (0.22–0.28 nm), indicating preserved global folding (Fig. 7A). Among ligand-bound complexes, the hinokinin-bound system showed the lowest average RMSD, whereas cubebin exhibited transient mid-trajectory fluctuations before stabilizing. Ligand RMSD analysis revealed marked differences in residence time (Fig. 7B). Matairesinol dissociated from the binding pocket after ~ 50 ns, and the control ligand dissociated after ~ 100 ns. Hinokinin remained bound until approximately 225 ns. In contrast, cubebin remained stably bound throughout the entire 300-ns simulation, indicating prolonged residence and enhanced complex stability.

Residue-level flexibility analysis (RMSF) showed localized increases in flexibility in regions surrounding the ligand-binding pocket (residues 249–259 and 408–412), most pronounced in the cubebin-bound complex (Fig. 7C), while the remainder of the protein exhibited similar dynamics across all systems.

### Cubebin forms a persistent interaction network with SF-1

Protein compactness, assessed by the radius of gyration, remained stable across all systems (1.85–1.87 nm), indicating no global unfolding (Fig. 7D). Cubebin induced a modest but sustained increase in Rg, consistent with stable pocket engagement and adaptive conformational rearrangements. Hydrogen-bond analysis demonstrated that hinokinin and matairesinol formed intermittent hydrogen bonds, while the control ligand showed only transient interactions (Supplementary Figure S1). In contrast, cubebin maintained 1–3 hydrogen bonds for the majority of the simulation, indicating a persistent interaction network that correlated with its prolonged residence time. Solvent-accessible surface area (SASA) analysis revealed higher solvent exposure for cubebin-bound complexes compared with apo SF-1 and hinokinin-bound systems, consistent with ligand-induced pocket rearrangements (Supplementary Figure S2).

#### Conformational landscapes and interaction energetics support cubebin stability

Principal component analysis (PCA) of backbone motions revealed distinct conformational sampling across systems (Supplementary Figure S3). Apo SF-1 occupied a compact conformational space, while matairesinol and control ligands induced broader sampling, indicative of increased flexibility. In contrast, the cubebin-bound complex showed a constrained and coherent conformational ensemble, suggesting stabilization of collective motions. Consistent with these findings, the free energy landscape (FEL) based on PC1 and PC2 indicated a more confined low-energy basin for the cubebin complex relative to other ligand-bound systems (Supplementary Figure S4). Representative trajectory snapshots for the cubebin complex are shown in Supplementary Figure S5. Interaction energy analysis further supported cubebin’s superior stability. Cubebin exhibited the most favorable total interaction energy (− 177.31 ± 16.9 kJ·mol⁻¹), exceeding those of hinokinin and the control ligand (Table 3). Binding free-energy calculations showed method-dependent differences: MM/PBSA yielded weakly unfavorable total binding energy due to high polar solvation penalties, whereas MM/GBSA predicted favorable binding (− 9.28 ± 0.24 kcal·mol⁻¹), dominated by van der Waals contributions. These results highlight the importance of hydrophobic interactions in stabilizing the SF-1–cubebin complex.

**Table 3:** Summary of post–molecular dynamics structural, dynamic, and interaction metrics for the apo protein and ligand-bound complexes. Values represent mean ± SD

Complex	H-bonds	SASA (nm²)	Total Interaction Energy (kJ/mol)
Apo (SF-1)	–	131.678 ± 2.515	–
4-Heptyloxyphenol (Control)	0.069 ± 0.293	138.301 ± 3.769	−105.93 ± 8.5
Cubebin	0.854 ± 0.680	139.297 ± 4.115	−177.31 ± 16.9
Hinokinin	0.195 ± 0.460	133.074 ± 4.109	−146.95 ± 9.6
Matairesinol	0.262 ± 0.626	139.082 ± 3.767	−101.30 ± 9.4

Collectively, docking, MD simulations, and free-energy analyses suggested that cubebin showed the most stable and persistent in silico interaction profile with SF-1 among the compounds tested. Its prolonged residence time, sustained hydrogen-bonding network, favourable interaction energy, and stabilized conformational dynamics distinguish it from both natural-product comparators and the known antagonist control. While these findings are exploratory and purely in silico, they nominate cubebin as a candidate ligand for future experimental validation of SF-1 modulation in HGSOC. These analyses do not demonstrate biological activity or therapeutic efficacy and are intended solely to assess structural feasibility.

## Discussion

In this study, we demonstrate that self-supervised contrastive learning applied to routine hematoxylin and eosin (H&E) whole-slide images can recover biologically meaningful but moderate transcriptomic variation in high-grade serous ovarian cancer (HGSOC). By integrating MoCo v2–derived tile embeddings with multi-output Random Forest regression, our framework predicted the expression of approximately 6,400 protein-coding genes with a performance comparable to or exceeding that reported in prior histology-based transcriptome inference studies [21, 24, 53], although direct comparison is limited by differences in tumor type, gene selection, and validation design. Notably, more than 300 genes showed stronger image–expression coupling (*r* > 0.44), indicating that measurable components of HGSOC transcriptional programs are reflected in standard diagnostic histopathology.

Compared with prior computational pathology studies, the present model shows moderate but biologically meaningful transcriptome-prediction performance. Earlier pan-cancer and tumor-specific studies have demonstrated that H&E-derived models can predict RNA-sequencing expression, molecular phenotypes, and clinically actionable alterations from histology, although performance varies substantially by tumor type, gene, endpoint, and validation design [18–24, 53, 54]. In ovarian cancer specifically, recent deep-learning studies have focused on direct prediction of platinum response or treatment-relevant transcriptomic states from histopathology rather than broad HGSOC-specific genome-wide expression inference [22, 23]. In this context, the present framework extends prior work by applying disease-focused self-supervised representation learning to HGSOC and by linking image-derived gene prioritization to independent RT-qPCR validation. Nevertheless, the observed correlation values remain below the level required for stand-alone patient-level clinical decision-making and should be interpreted as evidence of morphologically encoded transcriptional signal rather than definitive individual-level prediction.

These findings align with and extend prior work showing that deep learning models can infer molecular states, immune composition, and clinically relevant features directly from histomorphology [18–20, 54]. HGSOC is characterized by pronounced genomic instability, cellular plasticity, and a complex tumor–stroma interface [9]. The strong image-based predictability observed for transcription factors (e.g., *NR5A1*, SOX4, ZBTB7A), chromatin regulators (e.g., *SMARCB1, HCFC1, HMGA1*), and signaling or metabolic mediators (e.g., VEGFB, MAP2K2) suggests that core biological pathways such as steroidogenesis, chromatin remodeling, proliferation, and angiogenesis, produce consistent architectural signatures at the tissue level. Self-supervised contrastive learning is particularly well suited to capture these latent structures, as it leverages intrinsic morphological variability without requiring manual annotation or molecular supervision, enabling scalable deployment in settings where transcriptomic profiling is unavailable [25, 26].

A key contribution of this work is the integration of image-based virtual transcriptomics with independent experimental validation and structure-based modeling. Among the prioritized candidates, *NR5A1* (Steroidogenic Factor-1) emerged as a hypothesis-generating biomarker candidate because it combined strong image-based predictability, marked inter-patient expression heterogeneity, and significantly higher expression in platinum-responsive tumors in the independent RT-qPCR cohort. *NR5A1* is a nuclear receptor transcription factor with a central role in normal ovarian biology, where it regulates gonadal differentiation, folliculogenesis, and steroid hormone biosynthesis through coordinated control of steroidogenic gene expression and mitochondrial metabolic activity in granulosa and theca cells [55, 56]. Through these physiological functions, *NR5A1* integrates ovarian differentiation state with cellular metabolism and endocrine signaling. In the context of malignancy, dysregulation of developmental and steroidogenic transcriptional programs has been implicated in altered tumor differentiation states and metabolic reprogramming in ovarian cancer. Platinum response in epithelial ovarian cancer is strongly influenced by epithelial–mesenchymal transition and reprogrammed metabolic states [57, 58]. Accordingly, variation in *NR5A1* expression may reflect differences between more differentiated, metabolically “ovarian-like” tumor programs and more dedifferentiated, therapy-resistant phenotypes. This provides a biologically plausible explanation for both the strong image-based predictability of *NR5A1* and its association with platinum response observed in this study, without implying a direct causal role.

The availability of a resolved ligand-binding domain structure for *NR5A1/*SF-1 (PDB ID: 4QJR) further motivated its prioritization for downstream hypothesis generation. Structure-based molecular docking and molecular dynamics simulations were therefore employed strictly as proof-of-concept analyses to explore the structural feasibility of targeting *NR5A1* ligand binding, rather than to imply therapeutic efficacy. Among tested compounds, cubebin exhibited the most persistent binding behavior during extended simulations, characterized by prolonged residence within the ligand-binding pocket, sustained hydrogen-bonding interactions, and favorable interaction energetics relative to comparator ligands. While these in silico findings require biochemical and cellular validation, they illustrate how image-inferred transcriptomic signals can be directly coupled to rational target prioritization and early-stage therapeutic exploration. Conceptually, this framework differs from prior supervised histology-to-transcriptomics approaches, which rely on paired molecular labels during training [23]. Here, we demonstrate that self-supervised representation learning alone without transcriptomic supervision can support genome-wide expression prediction and uncover biologically and structurally actionable regulators. This distinction is critical for scalability, as it enables deployment in clinical and research settings where molecular profiling is limited, costly, or unavailable.

Several strengths of this study merit emphasis. Large-scale self-supervised pretraining on millions of unlabeled histology tiles enabled the model to learn robust, biologically grounded morphological representations without manual annotation. Multi-output regression allowed simultaneous modeling of thousands of genes, capturing shared structure among transcriptional programs rather than isolated targets. Rigorous patient-level cross-validation minimized information leakage and ensured robust performance estimates. Importantly, independent RT-qPCR validation provided biological corroboration of computational predictions, reinforcing the relevance of the image-derived signals.

Several limitations should be acknowledged. First, although the TCGA-OV dataset provided a large public resource for image–transcriptome modeling, external validation in independent multi-institutional WSI/RNA-seq cohorts was not available in the present study. Second, the RT-qPCR validation cohort was small (*n* = 10), with 4 responders and 6 non-responders, limiting statistical power and increasing uncertainty around response-associated differences. Third, the model’s moderate gene-expression prediction performance is not sufficient for individual-level clinical decision-making, and clinical translation would require prospective validation, calibration, and comparison with established molecular and clinicopathological predictors. Fourth, the RT-qPCR and structural analyses were exploratory and hypothesis-generating; no multiple-testing correction was applied to the RT-qPCR screen, and the docking and molecular dynamics simulations do not demonstrate biological activity or therapeutic efficacy. Fifth, although the model captured measurable transcriptomic signal from histology, many genes exhibited low predictability, likely reflecting weak morphological imprinting, spatial heterogeneity, or dominant contributions from non-tumor cell populations. Finally, differences in tissue processing, staining, scanner type, and tumor cellularity may affect model robustness and should be evaluated in future multi-center studies. Functional validation of candidate ligands and integration with spatial transcriptomics will be essential before these findings can be translated into clinical or therapeutic applications.

Future work should expand self-supervised pretraining and validation to multi-institutional datasets to enhance generalizability and explore transformer-based regressors capable of capturing broader spatial context. Integration with spatial transcriptomics and multiplex imaging will be essential for anchoring predicted gene-expression programs to specific microanatomical regions. Larger, clinically annotated HGSOC cohorts will be required to validate *NR5A1* and related markers in the context of platinum response and to assess their prognostic or predictive utility. Finally, systematic functional studies will also be necessary to evaluate candidate scaffolds and determine whether NR5A1 modulation has therapeutic relevance.

In summary, this study demonstrates that contrastive self-supervised learning applied to routine diagnostic histology captures measurable components of genome-wide transcriptional variation in HGSOC and enables biologically relevant biomarker prioritization. By integrating computational pathology, independent molecular validation, and proof-of-concept structure-based modeling, we outline a scalable, tissue-sparing framework for virtual transcriptomics and therapeutic hypothesis generation, highlighting the translational potential of AI-driven pathology in precision oncology. These findings support future validation of image-guided biomarker prioritization in larger, independent HGSOC cohorts, but they do not yet support individual-level clinical decision-making or therapeutic use without further experimental and prospective validation.

## Supplementary information

Supplementary Material 1. Supplementary Method S1: TCGA-HGSOC cohort selection and slide inclusion criteria. Supplementary Method S2: Whole-slide image preprocessing, tissue masking, and tile quality filtering. Supplementary Method S3: Self-supervised contrastive learning (MoCo v2) training details. Supplementary Method S4: Slide-level embedding aggregation and multi-output Random Forest regression. Supplementary Method S5: Cross-validation strategy and performance metrics. Supplementary Method S6: RT-qPCR experimental procedures (RNA extraction, cDNA synthesis, MIQE compliance). Supplementary Method S7: Candidate gene prioritization criteria. Supplementary Method S8: Molecular docking, molecular dynamics simulations, and free-energy calculations.

Supplementary Material 2. Supplementary Table-1: Candidate genes, RefSeq identifiers, and primers sequences details for RT-qPCR validation of candidate genes in the independent HGSOC cohort. Supplementary Table-2: MoCo v2 Architecture, training hyperparameters, and augmentation settings used for self-supervised representation learning. Supplementary Table-3: Hyperparameters and evaluation settings for multi-output Random Forest regression used for transcriptome prediction. Supplementary Table-4: Ligand library used for SF-1 molecular docking and corresponding docking scores. Supplementary Table-5: Top genes ranked by cross-validated Pearson correlation between predicted and observed RNA-sequencing expression values in the TCGA HGSOC cohort. Functional annotations reflect established biological roles relevant to ovarian cancer and tumor biology. Validation status indicates experimental assessment by RT-qPCR in the independent HGSOC cohort.

Supplementary Material 3. Supplementary Figure S1: Number of protein–ligand hydrogen bonds formed over the course of the simulations. (A) 4-Heptyloxyphenol (Control) (B) Cubebin (C) Hinokinin (D) Matairesinol. Supplementary Figure S2: Time evolution of solvation free energy (left) and solvent-accessible surface area (right) for SF-1–ligand complexes during molecular dynamics simulations. Supplementary Figure S3: Principal Component Analysis calculated based on the PC1 and PC2 (A) Apo protein (B) 4-Heptyloxyphenol (Control) (C) Cubebin (D) Hinokinin (E) Matairesinol. Supplementary Figure S4: Free Energy Landscape calculated based on the PC1 and PC2 (A) Apo protein (B) 4-Heptyloxyphenol (Control) (C) Cubebin (D) Hinokinin (E) Matairesinol. Supplementary Figure S5: VMD snapshots of the 4QJR-Cubebin complex during the 300ns production MD (A) Structure of cubebin (B) 0ns (C) 50ns (D) 100ns (E) 150ns (F) 200ns (G) 250ns (H) 300ns.

## Data Availability

TCGA-OV WSI and RNA-seq data are publicly available through the Genomic Data Commons (https://portal.gdc.cancer.gov/). RT-qPCR data and trained model embeddings will be deposited in Zenodo upon publication. Code supporting the computational pipeline for WSI preprocessing, self-supervised representation learning (MoCo v2), slide-level embedding construction, and multi-output transcriptome prediction is publicly available at: https://github.com/tsak1998/hgsoc-wsi-rna/. The repository includes scripts and documentation required to reproduce the analyses reported in this study.

## Abbreviations

HGSOC: High-grade serous ovarian cancer
H&E: Hematoxylin and eosin
WSI/WSIs: Whole-slide image(s)
TCGA-OV: The Cancer Genome Atlas Ovarian Serous Cystadenocarcinoma cohort
CPTAC: Clinical Proteomic Tumor Analysis Consortium
RECIST: Response Evaluation Criteria In Solid Tumors
RNA-seq: RNA-sequencing
MoCo v2: Momentum Contrast v2
RT-qPCR: Reverse transcription quantitative polymerase chain reaction
CV: Coefficient of variation
MD: Molecular dynamics
SF-1: Steroidogenic Factor-1
RMSD: Root mean square deviation
RMSF: Root mean square fluctuation
Rg: Radius of gyration
PCA: Principal component analysis
FEL: Free energy landscape
SASA: Solvent-accessible surface area
