# Supplementary material for: Deep learning-based prediction of gene expression from histopathology identifies NR5A1 as a candidate biomarker and druggable target in high-grade serous ovarian carcinoma
Source: J Ovarian Res. 2026 Jun 11;19:210. doi: 10.1186/s13048-026-02166-y (PMC13262399; doi:10.1186/s13048-026-02166-y)
Supplement: Supplementary file 1 — Supplementary Material 1. Supplementary Method S1: TCGA-HGSOC cohort selection and slide inclusion criteria. Supplementary Method S2: Whole-slide image preprocessing, tissue masking, and tile quality filtering. Supplementary Method S3: Self-supervised contrastive learning (MoCo v2) training details. Supplementary Method S4: Slide-level embedding aggregation and multi-output Random Forest regression. Supplementary Method S5: Cross-validation strategy and performance metrics. Supplementary Method S6: RT-qPCR experimental procedures (RNA extraction, cDNA synthesis, MIQE compliance). Supplementary Method S7: Candidate gene prioritization criteria. Supplementary Method S8: Molecular docking, molecular dynamics simulations, and free-energy calculations. [file 13048_2026_2166_MOESM1_ESM.docx]

**Supplementary Methods**

**Deep Learning-Based Prediction of Gene Expression from Histopathology Identifies *NR5A1* as a Candidate Biomarker and Druggable Target in High-Grade Serous Ovarian Carcinoma**

Prakash Lingasamy^1,2†^, Marta Ostrowska-Leśko^3†^, Pantelis Tsakalis^4^, Naisarg Patel^2,5^, Ilias Chamatidis^4,^ Sajitha Lulu Sudhakaran^5^, Joanna Kubik ^6^, Marcin Bobiński ^3^, Nikos Lagaros^4^, Andres Salumets^2,7,8*^, Vijayachitra Modhukur^2, 8*^

1. Laboratory of Precision and Nanomedicine, Institute of Biomedicine and Translational Medicine, University of Tartu, Tartu, 50411, Estonia
2. Celvia CC AS, 50411 Tartu, Estonia
3. Independent Laboratory of Translational Medicine, Chair of Medical Genetics, Medical University of Lublin, Radziwillowska 11, 20-080 Lublin, Poland
4. Inferesence(INFS), National Technical University of Athens, 15780 Athens, Greece.
5. Integrative Multiomics Lab, School of Bio Sciences and Technology, Vellore Institute of Technology, Vellore 632014, Tamil Nadu, India
6. Independent Medical Biology Unit, Medical University of Lublin, Jaczewskiego 8b, 20-093 Lublin, Poland
7. Division of Obstetrics and Gynaecology, Department of Clinical Science, Intervention and Technology (CLINTEC), Karolinska Institutet, and Karolinska University Hospital, 14152, Stockholm, Sweden
8. Department of Obstetrics and Gynecology, Institute of Clinical Medicine, University of Tartu, Tartu, 51014, Estonia.

†These authors contributed equally to this work and share first authorship

* To whom correspondence should be addressed.

* Correspondence:

Dr. Vijayachitra Modhukur, Department of Obstetrics and Gynaecology, Institute of Clinical Medicine, University of Tartu, 50406 Tartu, Estonia. E-mail: [vijayachitra.modhukur@ut.ee](mailto:vijayachitra.modhukur@ut.ee)

* Correspondence may also be addressed to

Prof. Andres Salumets, Division of Obstetrics and Gynaecology, Department of Clinical Science, Intervention and Technology (CLINTEC), Karolinska Institutet, and Karolinska University Hospital, 14152, Stockholm, Sweden. E-mail: [andres.salumets@ki.se](mailto:andres.salumets@ki.se)

**Supplementary Methods S1–S8**

- **S1:** Details of TCGA Data selection
- **S2:** Detailed WSI preprocessing thresholds
- **S3:** MoCo v2 architecture & hyperparameters
- **S4:** Tile aggregation equations
- **S5:** Random Forest hyperparameters
- **S6:** Full RT-qPCR protocol + primers
- **S7:** Docking & MD full protocols
- **S8:** Molecular Dynamics Simulations and Binding Free Energy Calculations

**Supplementary Methods S1. Details of TCGA Data selection**

***Whole-Slide Images (WSIs) Dataset:*** From the full TCGA-OV cohort, 1,371 diagnostic H&E slides were retrieved, comprising 1,208 tumor WSIs and 163 normal or adjacent non-tumor WSIs. All slides were downloaded in SVS format at the highest available resolution. For patients with multiple diagnostic WSIs, the most representative slide was selected based on tumor fraction, staining quality, and TCGA metadata and used for downstream modeling.

***RNA-Sequencing (RNA-seq) Data Processing***: Matched RNA-seq abundance values were obtained from the GDC as HTSeq-derived FPKM, FPKM-UQ, and, when available, RSEM quantifications. For all downstream modeling, gene expression values were log2-transformed using log2(FPKM-UQ + 1). Preprocessing of expression data included: removal of non–protein-coding genes; exclusion of genes with zero variance across all samples; harmonization of gene identifiers to Ensembl v98 annotations; and retention of genes present in at least 95% of samples. After filtering, approximately 6,400 protein-coding genes were retained as regression targets.

***Clinical and Pathological Metadata***: Clinical and pathological metadata were retrieved from the GDC and harmonized using Pan-Cancer TCGA clinical matrices. Variables extracted included age at diagnosis, FIGO stage, debulking (residual disease) status, platinum response classification (sensitive vs. resistant, when available), overall survival, and progression-free survival. All TCGA data were fully de-identified and publicly accessible; therefore, no additional patient consent or local Institutional Review Board (IRB) approval was required.

**Supplementary Methods S2 Whole-Slide Image Preprocessing and Tile Extraction**

All WSIs were processed in SVS format at the highest available resolution (20× or 40× objective magnification, depending on scanner) using the OpenSlide library (v3.4) (Goode et al., 2013). To standardize processing and reduce computational burden, WSIs were first downsampled to an effective 2.5× magnification for tissue mask generation. Tissue detection was performed on the downsampled image by converting to HSV color space and thresholding the saturation (S) channel using Otsu’s method to separate tissue from background glass regions (Otsu, 1979). Morphological closing and opening operations were applied to remove small spurious regions and noise. Only connected components of at least 50,000 pixels were retained to exclude debris and tiny fragments. The resulting binary tissue mask was then upsampled back to the target magnification and used to constrain tile extraction to tissue-rich regions.

Tiles of size 256 × 256 pixels were extracted at an effective 20× magnification. Non-overlapping tiling was used to simplify aggregation and reduce redundancy. Tiles were required to overlap the tissue mask by at least 60% of their area to be considered valid. No explicit stain normalization was applied; instead, stain variability was implicitly addressed through contrastive augmentations pipeline during self-supervised learning (Ruifrok & Johnston, 2001). Artifact removal was performed at the tile level. Tiles were excluded if they exhibited low sharpness (quantified by Laplacian variance below a predefined threshold), extreme brightness or darkness (mean intensity outside the 5th–95th percentile range), obvious pen or annotation marks (detected by heuristic thresholds on color channels), or insufficient tissue content (<60% overlap with the tissue mask). This ensured that only high-quality, tissue-rich tiles were used for representation learning.

Because individual WSIs can yield between ~5,000 and 30,000 valid tiles depending on tissue area, a subsampling strategy was adopted to balance computational cost and morphological diversity. After artifact filtering, up to 10,000 tiles per WSI were randomly sampled; when fewer than 10,000 valid tiles were available, all were retained. This strategy reduced overrepresentation of very large slides while preserving the heterogeneity of tumor architecture and microenvironment. To prevent information leakage between training and evaluation phases, tiles from the same slide were never split across different data folds, consistent with best practices in weakly supervised computational pathology (Campanella et al., 2019; Hou et al., 2016; Lu et al., 2021). All WSIs belonging to a given patient were assigned to the same fold. No supervised labels (clinical or molecular) were used during tile extraction or self-supervised training. All image preprocessing and tiling were implemented in Python 3.10 using OpenSlide, NumPy, OpenCV, and scikit-image, and executed on Slurm-managed high-performance computing (HPC) nodes (256–512 GB RAM, NVIDIA A100 GPUs). Depending on image size and tissue content, WSI preprocessing and tiling required approximately 2–6 minutes per slide.

**Supplementary Methods S3. Self-Supervised Learning Architecture and Training (MoCo v2)**

To learn discriminative morphological features from H&E WSIs without manual annotations, we adopted the MoCo v2 framework for self-supervised contrastive learning (Chen et al., 2020), building on the original Momentum Contrast formulation (He et al., 2020) and contrastive representation learning with InfoNCE (Oord et al., 2018). In this framework, two augmented views of each tile form a positive pair, while features from other tiles in a large memory queue act as negatives. The model is trained to map positive pairs close together in the embedding space while pushing negative pairs apart, thereby learning robust, biologically meaningful representations.

MoCo v2 was implemented with a ResNet-50 backbone as the query encoder (He et al., 2016). A two-layer projection head (2048 → 2048 → 128) mapped backbone features into a 128-dimensional space for the contrastive loss. A momentum-updated key encoder, parameterized as an exponential moving average of the query encoder with momentum coefficient m = 0.999, was used to compute features for negative samples. A dynamic dictionary queue of 65,536 keys was maintained to provide a large and consistent set of negatives across training iterations.

The model was trained using the InfoNCE contrastive loss, with temperature parameter τ = 0.2 (Oord et al., 2018). For each query embedding *q*, its corresponding positive key *k⁺* (another augmented view of the same tile) and a set of K negative keys (k⁻) from the queue were used to minimize:

$$\mathcal{L}_{\text{InfoNCE}}=-\log\frac{\exp(q\cdot k^{+}/\tau)}{\exp(q\cdot k^{+}/\tau)+\sum_{i=1}^{K} \exp(q\cdot k_{i}^{-}/\tau)}.$$

For each tile, two augmented variants were generated to form positive pairs. The augmentation pipeline followed recommended MoCo v2 settings with adaptations for histopathology, including random color jitter (brightness, contrast, saturation, hue), Gaussian blur, random horizontal and vertical flips, random rotations (0–90°), stain-preserving histogram perturbation, and random cropping (retaining 88–100% of the original tile area) (Chen et al., 2020; Ciga et al., 2022). These transformations promote invariance to staining variability, scanner differences, orientation, and minor structural perturbations.

Training was performed on millions of tiles extracted from TCGA HGSOC slides. Stochastic gradient descent (SGD) was used with an initial learning rate of 0.03 (cosine annealing schedule), momentum 0.9, and weight decay 1 × 10⁻⁴. The batch size was 512, and the model was trained for 200 epochs using mixed precision (FP16/FP32) on NVIDIA A100 GPUs. Tiles were shuffled at each epoch, and no slide- or patient-level labels were used during self-supervised training. After training, the ResNet-50 backbone of the query encoder was retained for downstream feature extraction, while the projection head used only for contrastive learning was discarded. A full summary of architectural parameters and hyperparameters is provided in Supplementary Table S2.

**Supplementary Methods S4. Slide-Level Embedding Construction**

Tile-level embeddings were extracted from the penultimate layer of the trained ResNet-50 backbone, before the contrastive projection head. Each tile was encoded into a 1,024-dimensional feature vector, capturing nuclear morphology, stromal architecture, tumor–stroma interfaces, and contextual microenvironmental patterns.

Because WSIs differ in tissue area and therefore in the number of available tiles, we aggregated tile-level embeddings into fixed-dimensional slide-level representations. Only tiles that passed all quality filters (tissue-rich, non-blurry, artifact-free) were included. For each WSI, up to 10,000 valid tiles were used. To construct a slide-level embedding, four element-wise summary statistics—mean, standard deviation, minimum, and maximum—were computed across the 1,024-dimensional embedding space. These four vectors were concatenated to form a 4,096-dimensional descriptor for each slide:

$$\text{WSI\_Embedding}=[\mu_{1},\ldots,\mu_{2048},\text{ }\sigma_{1},\ldots,\sigma_{2048},\text{ }\min_{1},\ldots,\min_{2048},\text{ }\max_{1},\ldots,\max_{2048}]$$

This statistical aggregation provides permutation-invariant representations (independent of tile ordering), captures both dominant and rare morphological patterns, and yields a compact, fixed-length feature vector suitable for multi-output regression, in line with prior weakly supervised WSI work (Ilse et al., 2018; Lu et al., 2021). Tile embeddings were stored as compressed NumPy arrays, and slide-level embeddings were archived as 4,096-dimensional vectors indexed by TCGA slide and patient barcodes. All embeddings were generated deterministically from fixed MoCo v2 checkpoints to ensure reproducibility.

**Supplementary Methods S5. Multi-Output Random Forest Regression and Evaluation**

To infer transcriptomic profiles directly from WSIs, we formulated gene-expression prediction as a multi-output regression problem. For each slide *i*, the 4,096-dimensional WSI embedding served as the input feature vector, and the matched log2-transformed FPKM-UQ expression values for approximately 6,400 protein-coding genes constituted the output vector. The goal was to learn a mapping *f: ℝ⁴⁰⁹⁶ → ℝᴳ*, where *G* is the number of retained genes.

A multi-output Random Forest (RF) regressor (scikit-learn v1.3) was used to jointly model all genes. RF was chosen for its robustness to high-dimensional input, ability to capture non-linear relationships between morphology and gene expression, and interpretable feature importance estimates (Breiman, 2001; Pedregosa et al., 2011). The model used 500 trees, a maximum depth of 30, a minimum of 2 samples per leaf, mean squared error as the splitting criterion, bootstrap sampling, and the default “sqrt” strategy for the number of features considered at each split. Multi-output mode was enabled so that a single ensemble of trees simultaneously modeled all gene targets, allowing correlated transcriptional programs to be learned, consistent with prior image-to-transcriptome approaches (Mondol et al., 2023; Pizurica et al., 2024; Schmauch et al., 2020).

Model performance was evaluated using 5-fold cross-validation. Folds were stratified by tumor versus normal status to maintain class balance, and slide-level partitioning ensured that WSIs and tiles from a single patient did not appear in both training and validation folds. Within each training fold, WSI embeddings were z-score normalized, and the corresponding parameters were applied to the held-out validation fold. Hyperparameters were fixed a priori and not tuned post hoc to avoid optimistic bias. Predicted gene-expression values for each slide were obtained by applying the trained RF model to the corresponding WSI embedding. Genes with unstable predictions (low variance across folds) were flagged and excluded from downstream interpretive analyses.

To explore which morphological features contributed to gene-expression prediction, feature importance scores were computed from the trained RF model. Importance values were aggregated across trees to yield a 4,096-dimensional importance profile for each gene. Genes were ranked by performance metrics (e.g., correlation between predicted and observed expression) and by the strength and robustness of their feature importance profiles. This ranking was later used to prioritize candidate genes, including transcription factors such as *NR5A1*, for experimental validation and molecular docking. Full model configuration and evaluation settings are summarized in Supplementary Table S3.

**Supplementary Methods S6. RT-qPCR Experimental Procedures and Quality Control**

***RT-qPCR Gene Expression Analysis*:** Total RNA was extracted from HGSOC tissue samples preserved in RNAlater and stored at –80 °C. RNA isolation was performed using TRIzol™ Reagent (Invitrogen, Carlsbad, CA, USA) according to the manufacturer’s instructions, following the protocol (Chomczynski & Sacchi, 2006). RNA concentration and purity were assessed spectrophotometrically using a NanoDrop 2000 (Thermo Fisher Scientific, Waltham, MA, USA), and RNA integrity was verified by agarose gel electrophoresis.

For cDNA synthesis, 1 µg of total RNA per sample was reverse-transcribed using the NG dART RT-PCR kit (EURx, Gdańsk, Poland) on a Mastercycler Gradient thermocycler (Eppendorf, Hamburg, Germany) following the manufacturer's protocol Thermal cycling conditions were as follows: 10 minutes at 25 °C, 50 minutes at 50 °C, and a final step at 85 °C for 5 minutes. RT-qPCR was performed in triplicate using SG/ROX SYBR Green Master Mix (EURx, Gdańsk, Poland) on an Applied Biosystems 7500 Fast Real-Time PCR system (Foster City, CA, USA). The cycling conditions included an initial denaturation step at 95 °C for 2 min, followed by 40 cycles of denaturation at 95 °C for 10 s, annealing at 60 °C for 30 s, and extension at 72 °C for 30 s. A melting curve analysis was conducted to confirm amplicon specificity.

Eighteen candidate genes were analyzed: VEGFB, SMARCB1 (SNF5), NR5A1, SKI, MEK2, ZBTB7A, DGKZ, USP15, MMLT1, ARMC8, HCFC1, STRN4, SEC22L2, HMGA1, SOX4, FOXO1, CDK2AP2, and the reference genes ACTB and RNA18SN5. Primer sequences and RefSeq identifiers are provided in Table 2. RT-qPCR reactions were performed in triplicate. Expression levels were quantified using the 2⁻ΔCt method, with ΔCt defined as Ct(target gene) − Ct (geometric mean of ACTB and RNA18SN5), following MIQE recommendations and standard RT-qPCR analysis practice (Bustin et al., 2025; Livak & Schmittgen, 2001). NR5A1 (also known as SF-1) was quantified using a single primer set targeting the canonical transcript and is reported only once throughout the manuscript

Two endogenous reference genes, 18S rRNA (RNA18SN5) and β-actin (ACTB), were used for normalization to minimize technical variability. The final expression value for each sample and gene was computed as the mean of three technical replicates. Samples or reactions with aberrant melt curves, Ct > 35 for reference genes, or high replicate variability were excluded and repeated when necessary.

For each gene, the coefficient of variation (CV = standard deviation / mean) of 2⁻ΔCt values across the 10 samples was calculated to quantify inter-sample expression heterogeneity. High CV values were used to prioritize genes exhibiting substantial biological variability. Expression levels were compared between responders and non-responders using unpaired t-tests, and associations with FIGO stage and grade were explored descriptively. These analyses were used to guide candidate selection for molecular docking and further functional interpretation. Candidate genes, RefSeq identifiers, and primers sequences details are provided in Supplementary Table S1.

**Supplementary Methods S7. Candidate selection and Molecular Docking**

**Candidate Selection for Molecular Docking:** Genes were shortlisted for docking studies based on three criteria: (i) high inter-sample variability in RT-qPCR expression (high CV); (ii) statistically or biologically meaningful differences between responders and non-responders; and (iii) availability of an experimentally resolved protein structure in the Protein Data Bank (PDB) with a well-defined ligand-binding pocket amenable to small-molecule docking. NR5A1 (Steroidogenic Factor-1) was selected as the primary candidate due to its high expression variability, significant association with platinum response, established role in steroidogenic and endocrine signaling, and the availability of a ligand-binding domain structure (PDB ID: 4QJR). SMARCB1(SNF5; PDB ID: 4HQC) was also considered due to its association with tumor grade; however, it was not pursued in docking studies due to the lack of a canonical small-molecule binding pocket. All docking and molecular dynamics analyses were performed exclusively to assess structural feasibility and ligand–protein interaction stability and were not intended to predict therapeutic efficacy or clinical activity.

**Structure-Based Molecular Docking:** The crystallographic structure of the ligand-binding domain of NR5A1 was retrieved from the Protein Data Bank (PDB ID: 4QJR). Potential ligand-binding pockets were identified using the CASTpFold server, which detects surface-accessible cavities and internal pockets based on geometric and topological criteria (Ye et al., 2024). A curated library of candidate anti-cancer compounds was assembled from published literature (Supplementary Table S4), focusing on small molecules with reported biological activity relevant to nuclear receptors or cancer-associated signaling pathways. Virtual screening of these compounds was performed using AutoDock Vina, which estimates binding affinity through a stochastic global optimization algorithm combined with an empirical scoring function (Eberhardt et al., 2021). Docking grids were centered on the predicted NR5A1 ligand-binding pocket, with grid dimensions chosen to fully encompass the cavity and adjacent interaction regions. For each ligand, multiple binding poses were generated, and predicted binding affinities (kcal/mol) were recorded. Ligands were ranked based on their lowest-energy docking pose, and top-scoring candidates were shortlisted for further analysis. Three compounds (CHEMBL compound IDs: 25641, 4131581, and 121345502) were included as reference controls based on previously reported antagonist activity against NR5A1 or related nuclear receptors in biochemical assays (ChEMBL IDs: CHEMBL1767443 and CHEMBL3776839) (Hirata et al., 2016; Whitby et al., 2011). These controls were used to benchmark docking performance and binding mode plausibility.

**Supplementary Methods S8. Molecular Dynamics Simulations and Binding Free Energy Calculations**

Molecular dynamics (MD) simulations were performed to evaluate the structural stability and dynamic behavior of NR5A1 in both apo form and in complex with selected ligands. System preparation and simulation protocols were implemented in GROMACS (version 2023.2) following established best practices for biomolecular simulations (Abraham et al., 2015; Lemkul, 2019). Protein and protein–ligand complexes were solvated in an explicit water box, neutralized with counterions, and subjected to energy minimization prior to equilibration. The CHARMM36 force field was used to parameterize the protein (Soteras Gutierrez et al., 2016), while ligand parameters were generated using the CHARMM General Force Field (CGenFF) (Vanommeslaeghe et al., 2012). Each system underwent sequential equilibration under constant volume (NVT) and constant pressure (NPT) conditions before production runs. Production MD simulations were conducted for 300 ns for each system.

MD simulations were not used for ranking compounds clinically. Trajectory analyses were performed using GROMACS tools to assess structural stability, conformational dynamics, and protein–ligand interactions. Root-mean-square deviation (RMSD), root-mean-square fluctuation (RMSF), and interaction persistence metrics were computed. Trajectories were visualized using Visual Molecular Dynamics (VMD), and summary plots were generated in R (Humphrey et al., 1996). Binding free energies were estimated using the Molecular Mechanics Poisson–Boltzmann Surface Area (MM/PBSA) and Molecular Mechanics Generalized Born Surface Area (MM/GBSA) approaches implemented via the gmx_MMPBSA package (Valdes-Tresanco et al., 2021). To ensure analysis of equilibrated states, the final 20 ns of each trajectory were excluded. Frames were extracted every 200 ps from the equilibrated portion of the trajectory, yielding 100 frames per system for free energy calculations. Mean binding free energies and standard deviations were calculated across extracted frames. Summary docking and MD results are provided in Supplementary Tables S5 and S6.

**Reference:**

Abraham, M. J., Murtola, T., Schulz, R., Páll, S., Smith, J. C., Hess, B., & Lindahl, E. (2015). GROMACS: High performance molecular simulations through multi-level parallelism from laptops to supercomputers. *SoftwareX*, *1-2*, 19-25. <https://doi.org/10.1016/j.softx.2015.06.001>

Breiman, L. (2001). Random Forests. *Machine Learning 2001 45:1*, *45*(1). <https://doi.org/10.1023/A:1010933404324>

Bustin, S. A., Ruijter, J. M., van den Hoff, M. J. B., Kubista, M., Pfaffl, M. W., Shipley, G. L., Tran, N., Rodiger, S., Untergasser, A., Mueller, R., Nolan, T., Milavec, M., Burns, M. J., Huggett, J. F., Vandesompele, J., & Wittwer, C. T. (2025). MIQE 2.0: Revision of the Minimum Information for Publication of Quantitative Real-Time PCR Experiments Guidelines. *Clin Chem*. <https://doi.org/10.1093/clinchem/hvaf043>

Campanella, G., Hanna, M. G., Geneslaw, L., Miraflor, A., Werneck Krauss Silva, V., Busam, K. J., Brogi, E., Reuter, V. E., Klimstra, D. S., & Fuchs, T. J. (2019). Clinical-grade computational pathology using weakly supervised deep learning on whole slide images. *Nat Med*, *25*(8), 1301-1309. <https://doi.org/10.1038/s41591-019-0508-1>

Chen, X., Fan, H., Girshick, R., & He, K. (2020). Improved Baselines with Momentum Contrastive Learning. <https://doi.org/10.48550/arXiv.2003.04297>

Chomczynski, P., & Sacchi, N. (2006). The single-step method of RNA isolation by acid guanidinium thiocyanate-phenol-chloroform extraction: twenty-something years on. *Nat Protoc*, *1*(2), 581-585. <https://doi.org/10.1038/nprot.2006.83>

Ciga, O., Xu, T., & Martel, A. L. (2022). Self supervised contrastive learning for digital histopathology. *Machine Learning with Applications*, *7*. <https://doi.org/ARTN> 100198

10.1016/j.mlwa.2021.100198

Eberhardt, J., Santos-Martins, D., Tillack, A. F., & Forli, S. (2021). AutoDock Vina 1.2.0: New Docking Methods, Expanded Force Field, and Python Bindings. *J Chem Inf Model*, *61*(8), 3891-3898. <https://doi.org/10.1021/acs.jcim.1c00203>

Goode, A., Gilbert, B., Harkes, J., Jukic, D., & Satyanarayanan, M. (2013). OpenSlide: A vendor-neutral software foundation for digital pathology. *J Pathol Inform*, *4*, 27. <https://doi.org/10.4103/2153-3539.119005>

He, K. M., Fan, H. Q., Wu, Y. X., Xie, S. N., & Girshick, R. (2020). Momentum Contrast for Unsupervised Visual Representation Learning. *2020 Ieee/Cvf Conference on Computer Vision and Pattern Recognition (Cvpr 2020)*, 9726-9735. <https://doi.org/10.1109/Cvpr42600.2020.00975>

He, K. M., Zhang, X. Y., Ren, S. Q., & Sun, J. (2016). Deep Residual Learning for Image Recognition. *2016 Ieee Conference on Computer Vision and Pattern Recognition (Cvpr)*, 770-778. <https://doi.org/10.1109/Cvpr.2016.90>

Hirata, K., Kotoku, M., Seki, N., Maeba, T., Maeda, K., Hirashima, S., Sakai, T., Obika, S., Hori, A., Hase, Y., Yamaguchi, T., Katsuda, Y., Hata, T., Miyagawa, N., Arita, K., Nomura, Y., Asahina, K., Aratsu, Y., Kamada, M.,…Shiozaki, M. (2016). SAR Exploration Guided by LE and Fsp(3): Discovery of a Selective and Orally Efficacious RORgamma Inhibitor. *ACS Med Chem Lett*, *7*(1), 23-27. <https://doi.org/10.1021/acsmedchemlett.5b00253>

Hou, L., Samaras, D., Kurc, T. M., Gao, Y., Davis, J. E., & Saltz, J. H. (2016). Patch-based Convolutional Neural Network for Whole Slide Tissue Image Classification. *Proc IEEE Comput Soc Conf Comput Vis Pattern Recognit*, *2016*, 2424-2433. <https://doi.org/10.1109/CVPR.2016.266>

Humphrey, W., Dalke, A., & Schulten, K. (1996). VMD: visual molecular dynamics. *J Mol Graph*, *14*(1), 33-38, 27-38. <https://doi.org/10.1016/0263-7855(96)00018-5>

Ilse, M., Tomczak, J. M., & Welling, M. (2018). Attention-based Deep Multiple Instance Learning. *International Conference on Machine Learning, Vol 80*, *80*. <https://doi.org/10.48550/arXiv.1802.04712>

Lemkul, J. A. (2019). From Proteins to Perturbed Hamiltonians: A Suite of Tutorials for the GROMACS-2018 Molecular Simulation Package [Article v1.0]. *Living Journal of Computational Molecular Science*, *1*(1). <https://doi.org/10.33011/livecoms.1.1.5068>

Livak, K. J., & Schmittgen, T. D. (2001). Analysis of relative gene expression data using real-time quantitative PCR and the 2(-Delta Delta C(T)) Method. *Methods*, *25*(4), 402-408. <https://doi.org/10.1006/meth.2001.1262>

Lu, M. Y., Williamson, D. F. K., Chen, T. Y., Chen, R. J., Barbieri, M., & Mahmood, F. (2021). Data-efficient and weakly supervised computational pathology on whole-slide images. *Nat Biomed Eng*, *5*(6), 555-570. <https://doi.org/10.1038/s41551-020-00682-w>

Mondol, R. K., Millar, E. K. A., Graham, P. H., Browne, L., Sowmya, A., & Meijering, E. (2023). hist2RNA: An Efficient Deep Learning Architecture to Predict Gene Expression from Breast Cancer Histopathology Images. *Cancers (Basel)*, *15*(9). <https://doi.org/10.3390/cancers15092569>

Oord, A. v. d., Li, Y., & Vinyals, O. (2018). Representation Learning with Contrastive Predictive Coding. <https://doi.org/10.48550/arXiv.1807.03748>

Otsu, N. (1979). A Threshold Selection Method from Gray-Level Histograms. *IEEE Transactions on Systems, Man, and Cybernetics*, *9*(1), 62-66. <https://doi.org/10.1109/tsmc.1979.4310076>

Pedregosa, F., Varoquaux, G., Gramfort, A., Michel, V., Thirion, B., Grisel, O., Blondel, M., Prettenhofer, P., Weiss, R., Dubourg, V., Vanderplas, J., Passos, A., Cournapeau, D., Brucher, M., Perrot, M., & Duchesnay, E. (2011). Scikit-learn: Machine Learning in Python. *Journal of Machine Learning Research*, *12*, 2825-2830. <https://doi.org/10.48550/arXiv.1201.0490>

Pizurica, M., Zheng, Y., Carrillo-Perez, F., Noor, H., Yao, W., Wohlfart, C., Vladimirova, A., Marchal, K., & Gevaert, O. (2024). Digital profiling of gene expression from histology images with linearized attention. *Nat Commun*, *15*(1), 9886. <https://doi.org/10.1038/s41467-024-54182-5>

Ruifrok, A. C., & Johnston, D. A. (2001). Quantification of histochemical staining by color deconvolution. *Anal Quant Cytol Histol*, *23*(4), 291-299. <https://www.ncbi.nlm.nih.gov/pubmed/11531144>

Schmauch, B., Romagnoni, A., Pronier, E., Saillard, C., Maille, P., Calderaro, J., Kamoun, A., Sefta, M., Toldo, S., Zaslavskiy, M., Clozel, T., Moarii, M., Courtiol, P., & Wainrib, G. (2020). A deep learning model to predict RNA-Seq expression of tumours from whole slide images. *Nat Commun*, *11*(1), 3877. <https://doi.org/10.1038/s41467-020-17678-4>

Soteras Gutierrez, I., Lin, F. Y., Vanommeslaeghe, K., Lemkul, J. A., Armacost, K. A., Brooks, C. L., 3rd, & MacKerell, A. D., Jr. (2016). Parametrization of halogen bonds in the CHARMM general force field: Improved treatment of ligand-protein interactions. *Bioorg Med Chem*, *24*(20), 4812-4825. <https://doi.org/10.1016/j.bmc.2016.06.034>

Valdes-Tresanco, M. S., Valdes-Tresanco, M. E., Valiente, P. A., & Moreno, E. (2021). gmx_MMPBSA: A New Tool to Perform End-State Free Energy Calculations with GROMACS. *J Chem Theory Comput*, *17*(10), 6281-6291. <https://doi.org/10.1021/acs.jctc.1c00645>

Vanommeslaeghe, K., Raman, E. P., & MacKerell, A. D., Jr. (2012). Automation of the CHARMM General Force Field (CGenFF) II: assignment of bonded parameters and partial atomic charges. *J Chem Inf Model*, *52*(12), 3155-3168. <https://doi.org/10.1021/ci3003649>

Whitby, R. J., Stec, J., Blind, R. D., Dixon, S., Leesnitzer, L. M., Orband-Miller, L. A., Williams, S. P., Willson, T. M., Xu, R., Zuercher, W. J., Cai, F., & Ingraham, H. A. (2011). Small molecule agonists of the orphan nuclear receptors steroidogenic factor-1 (SF-1, NR5A1) and liver receptor homologue-1 (LRH-1, NR5A2). *J Med Chem*, *54*(7), 2266-2281. <https://doi.org/10.1021/jm1014296>

Ye, B., Tian, W., Wang, B., & Liang, J. (2024). CASTpFold: Computed Atlas of Surface Topography of the universe of protein Folds. *Nucleic Acids Res*, *52*(W1), W194-W199. <https://doi.org/10.1093/nar/gkae415>
