# Supplementary material for: Deep learning-based prediction of gene expression from histopathology identifies NR5A1 as a candidate biomarker and druggable target in high-grade serous ovarian carcinoma
Source: J Ovarian Res. 2026 Jun 11;19:210. doi: 10.1186/s13048-026-02166-y (PMC13262399; doi:10.1186/s13048-026-02166-y)
Supplement: Supplementary file 3 — Supplementary Material 3. Supplementary Figure S1: Number of protein–ligand hydrogen bonds formed over the course of the simulations. (A) 4-Heptyloxyphenol (Control) (B) Cubebin (C) Hinokinin (D) Matairesinol. Supplementary Figure S2: Time evolution of solvation free energy (left) and solvent-accessible surface area (right) for SF-1–ligand complexes during molecular dynamics simulations. Supplementary Figure S3: Principal Component Analysis calculated based on the PC1 and PC2 (A) Apo protein (B) 4-Heptyloxyphenol (Control) (C) Cubebin (D) Hinokinin (E) Matairesinol. Supplementary Figure S4: Free Energy Landscape calculated based on the PC1 and PC2 (A) Apo protein (B) 4-Heptyloxyphenol (Control) (C) Cubebin (D) Hinokinin (E) Matairesinol. Supplementary Figure S5: VMD snapshots of the 4QJR-Cubebin complex during the 300ns production MD (A) Structure of cubebin (B) 0ns (C) 50ns (D) 100ns (E) 150ns (F) 200ns (G) 250ns (H) 300ns. [file 13048_2026_2166_MOESM3_ESM.docx]

**Deep Learning-Based Prediction of Gene Expression from Histopathology Identifies *NR5A1* as a Candidate Biomarker and Druggable Target in High-Grade Serous Ovarian Carcinoma**

Prakash Lingasamy^1,2†^, Marta Ostrowska-Leśko^3†^, Pantelis Tsakalis^4^, Naisarg Patel^2,5^, Ilias Chamatidis^4,^ Sajitha Lulu Sudhakaran^5^, Joanna Kubik ^6^, Marcin Bobiński ^3^, Nikos Lagaros^4^, Andres Salumets^2,7,8*^, Vijayachitra Modhukur^2, 8*^

1. Laboratory of Precision and Nanomedicine, Institute of Biomedicine and Translational Medicine, University of Tartu, Tartu, 50411, Estonia
2. Celvia CC AS, 50411 Tartu, Estonia
3. Independent Laboratory of Translational Medicine, Chair of Medical Genetics, Medical University of Lublin, Radziwillowska 11, 20-080 Lublin, Poland
4. Inferesence(INFS), National Technical University of Athens, 15780 Athens, Greece.
5. Integrative Multiomics Lab, School of Bio Sciences and Technology, Vellore Institute of Technology, Vellore 632014, Tamil Nadu, India
6. Independent Medical Biology Unit, Medical University of Lublin, Jaczewskiego 8b, 20-093 Lublin, Poland
7. Division of Obstetrics and Gynaecology, Department of Clinical Science, Intervention and Technology (CLINTEC), Karolinska Institutet, and Karolinska University Hospital, 14152, Stockholm, Sweden
8. Department of Obstetrics and Gynecology, Institute of Clinical Medicine, University of Tartu, Tartu, 51014, Estonia.

†These authors contributed equally to this work and share first authorship

* To whom correspondence should be addressed.

* Correspondence:

Dr. Vijayachitra Modhukur, Department of Obstetrics and Gynaecology, Institute of Clinical Medicine, University of Tartu, 50406 Tartu, Estonia. E-mail: [vijayachitra.modhukur@ut.ee](mailto:vijayachitra.modhukur@ut.ee)

* Correspondence may also be addressed to

Prof. Andres Salumets, Division of Obstetrics and Gynaecology, Department of Clinical Science, Intervention and Technology (CLINTEC), Karolinska Institutet, and Karolinska University Hospital, 14152, Stockholm, Sweden. E-mail: [andres.salumets@ki.se](mailto:andres.salumets@ki.se)

**Supplementary Figure S1:** Number of protein–ligand hydrogen bonds formed over the course of the simulations. (A) 4-Heptyloxyphenol (Control) (B) Cubebin (C) Hinokinin (D) Matairesinol


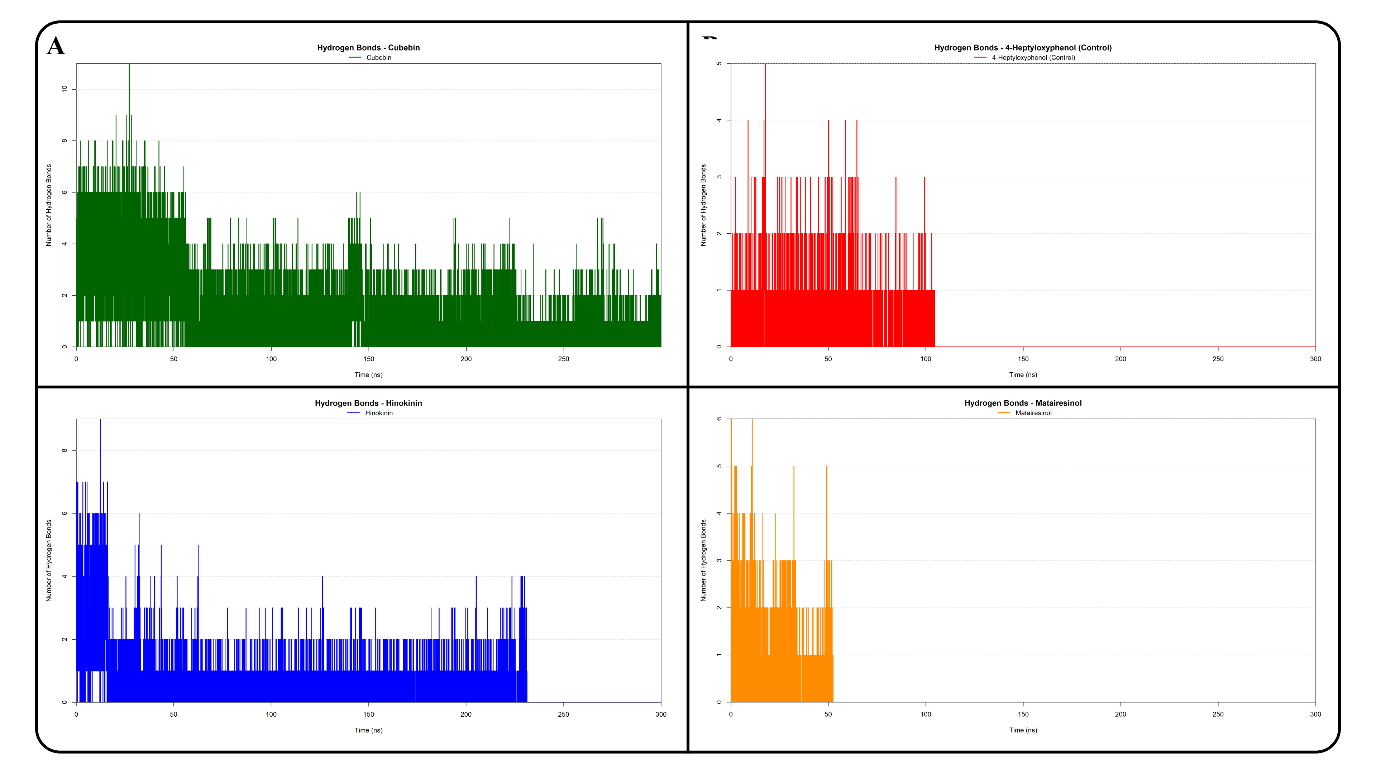


**Supplementary Figure S2:** Time evolution of solvation free energy (left) and solvent-accessible surface area (right) for SF-1–ligand complexes during molecular dynamics simulations


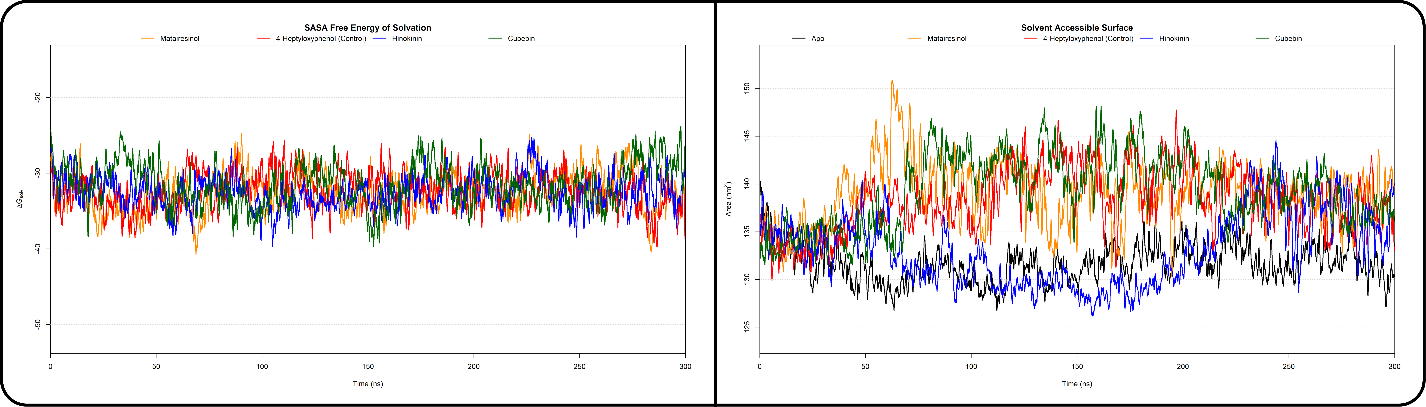


**Supplementary Figure S3:** Principal Component Analysis calculated based on the PC1 and PC2 (A) Apo protein (B) 4-Heptyloxyphenol (Control) (C) Cubebin (D) Hinokinin (E) Matairesinol


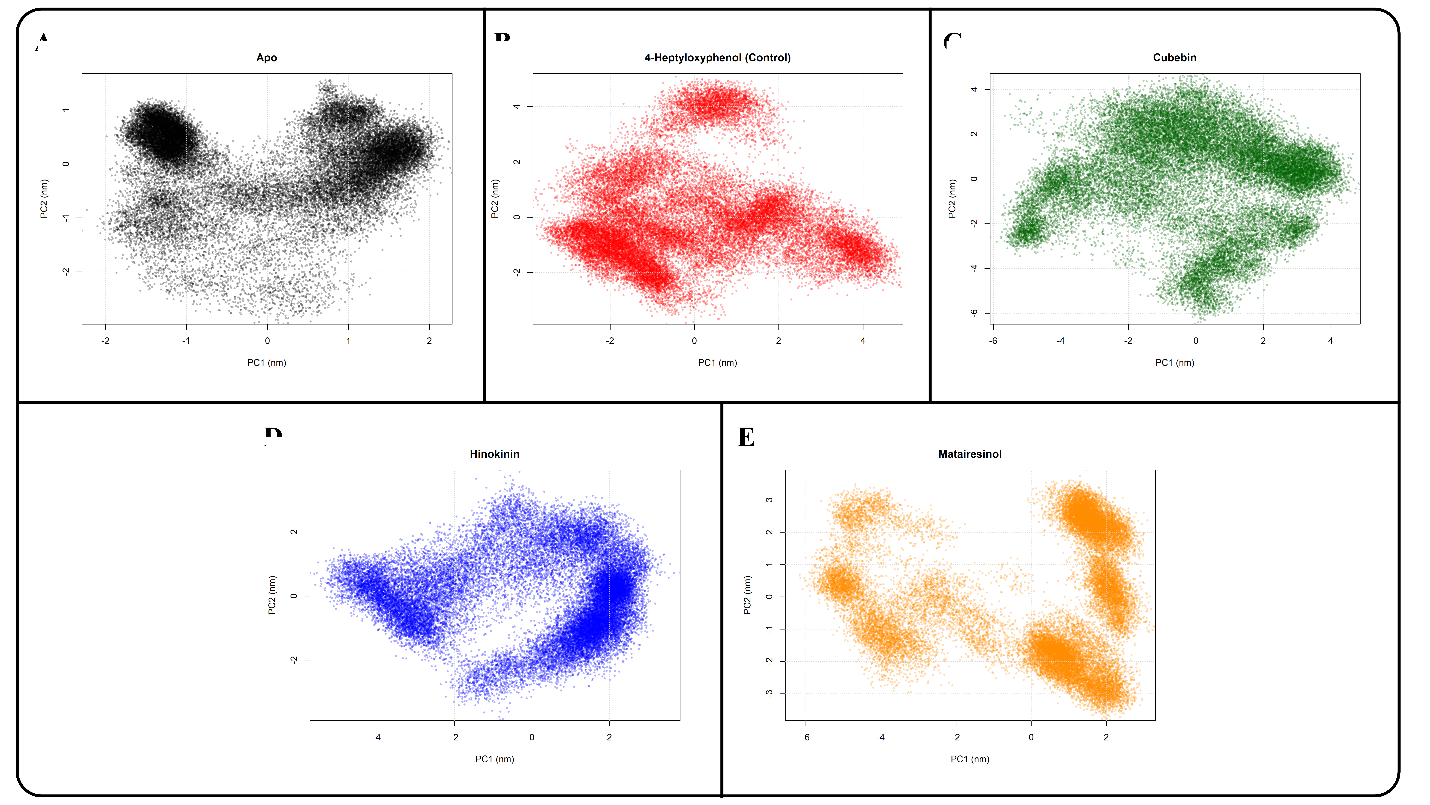


**Supplementary Figure S4:** Free Energy Landscape calculated based on the PC1 and PC2 (A) Apo protein (B) 4-Heptyloxyphenol (Control) (C) Cubebin (D) Hinokinin (E) Matairesinol

*
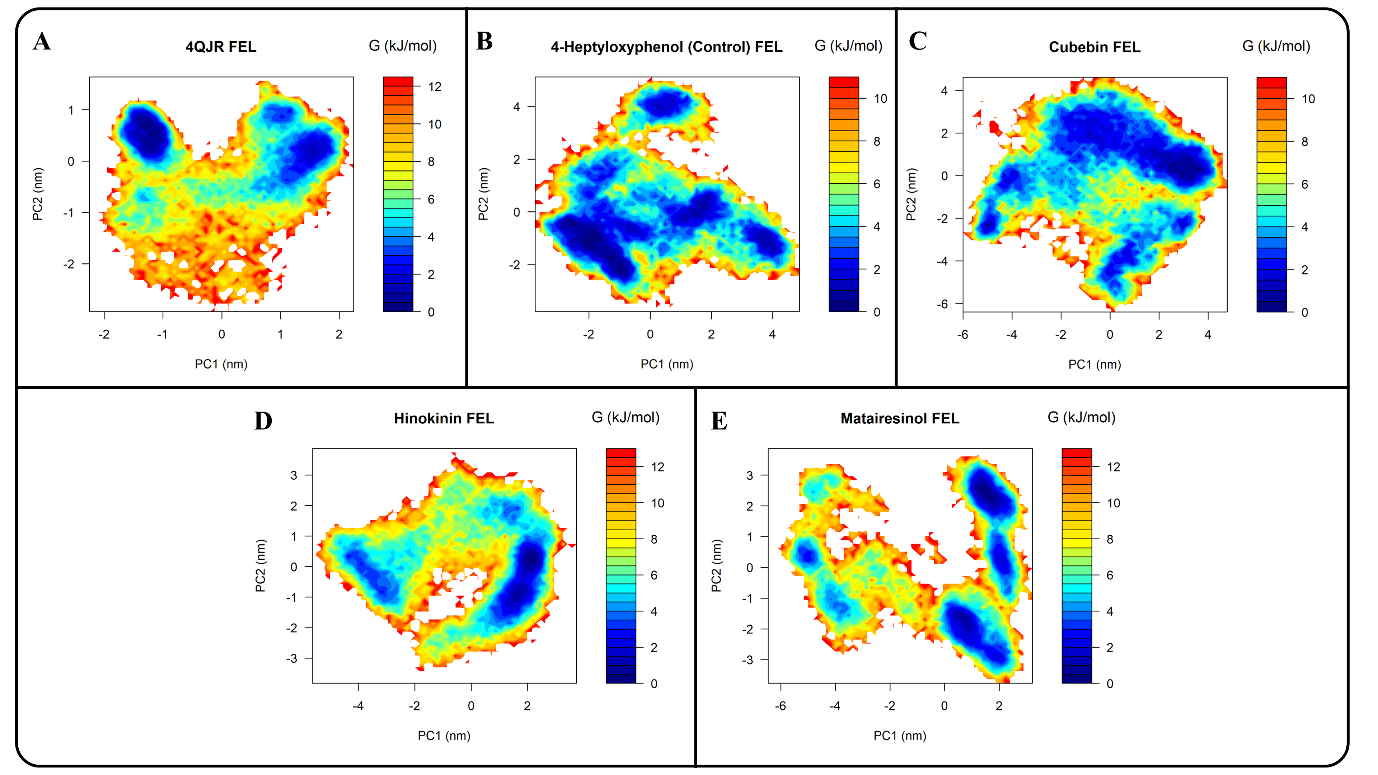
*

**Supplementary Figure S5:** VMD snapshots of the 4JQR-Cubebin complex during the 300ns production MD (A) Structure of cubebin (B) 0ns (C) 50ns (D) 100ns (E) 150ns (F) 200ns (G) 250ns (H) 300ns

*
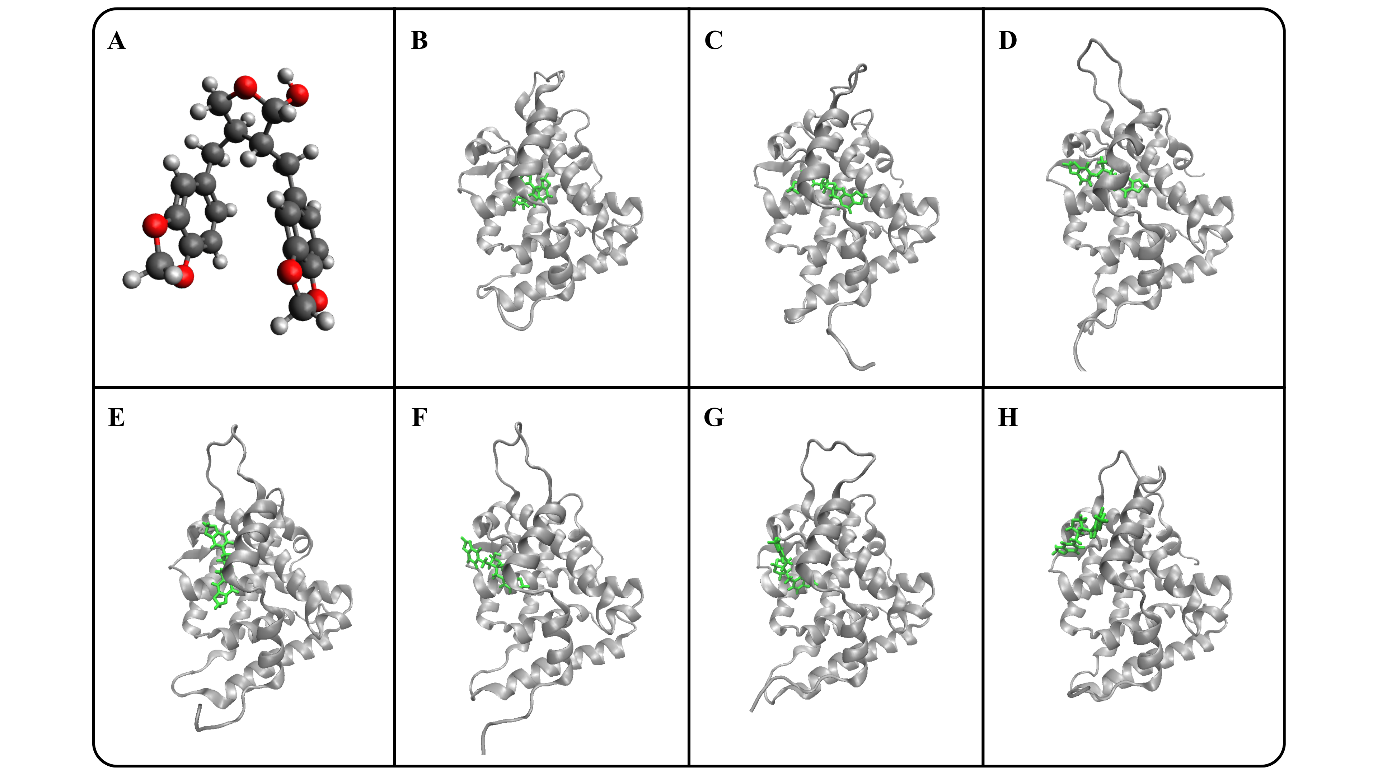
*
